# Comprehensive personalized ankle joint shape analysis of children with cerebral palsy from pediatric MRI

**DOI:** 10.3389/fbioe.2022.1059129

**Published:** 2022-11-25

**Authors:** Yue Cheng, Rodolphe Bailly, Claire Scavinner-Dorval, Benjamin Fouquet, Bhushan Borotikar, Douraied Ben Salem, Sylvain Brochard, François Rousseau

**Affiliations:** ^1^ IMT Atlantique, LaTIM U1101 INSERM, Brest, France; ^2^ Fondation Ildys, LaTIM U1101 INSERM, Brest, France; ^3^ Symbiosis Centre for Medical Image Analysis, Pune, India; ^4^ CHU, UBO, LaTIM U1101 INSERM, Brest, France

**Keywords:** shape analysis, ankle joint, MRI, cerebral palsy, morphometry

## Abstract

Cerebral palsy, a common physical disability in childhood, often causes abnormal patterns of movement and posture. To better understand the pathology and improve rehabilitation of patients, a comprehensive bone shape analysis approach is proposed in this article. First, a group analysis is performed on a clinical MRI dataset using two state-of-the-art shape analysis methods: ShapeWorks and a voxel-based method relying on Advanced Normalization Tools (ANTs) registration. Second, an analysis of three bones of the ankle is done to provide a complete view of the ankle joint. Third, a bone shape analysis is carried out at subject level to highlight variability patterns for personnalized understanding of deformities.

## 1 Introduction

**FIGURE 1:**
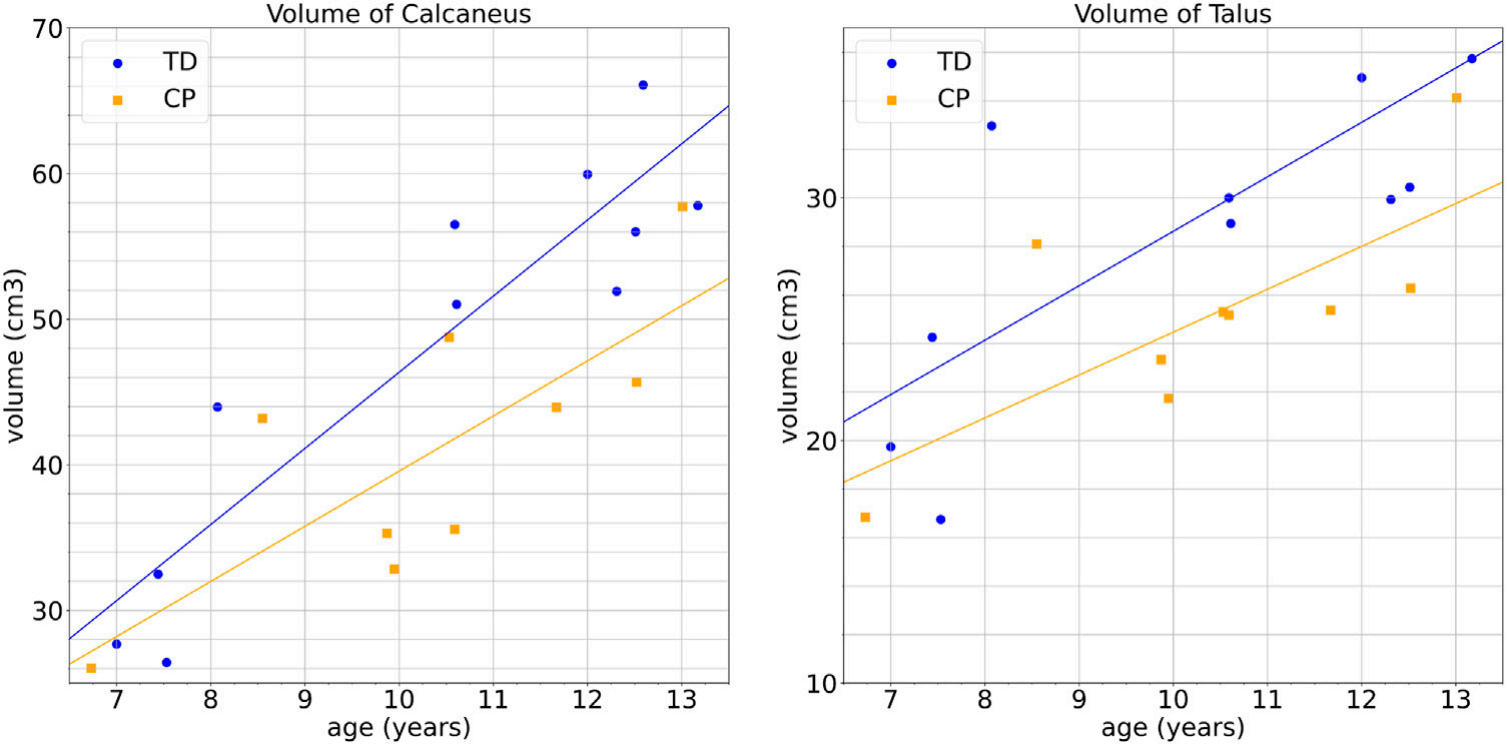
Bone and growth cartilage volumes of children with CP and typical developping (TD) children according to age: left: calcaneus, right: talus.

Cerebral palsy (CP) is the most common physical disability in childhood, affecting 2.1 out of every 1,000 individuals born. As a result of this non-progressive condition, abnormal movements and postures may occur, as well as impairments in cognitive function and sensory function, such as equinus, the most common musculo-skeletal deformity in children with CP (Davis et al. (1991); Metaxiotis et al. (2002)). In children with bilateral cerebral palsy, equinus prevalence is 83.3% and tends to increase with age. It manifests poor muscle control and weakness around the ankle and foot, leading to abnormal gait patterns and bone deformations during growth (Perry et al. (1974)). It has been reported that muscle morphology has been assessed (Schlegl et al. (2019)), but very little information is available on changes of bone structure. Pediatric MRI studies remain difficult to conduct due to complex acquisition settings (Mitchell et al. (2018); Makki et al. (2019)), however, according to our preliminary analysis, volume differences can be observed between controls and children with CP (see Figure 1 for volume measurements of calcaneus and talus). It suggests that a more detailed approach to studying the morphology of the ankle joint could lead to a better understanding of CP and personalized management of patients.

To determine whether an intervention is effective, studies report mean differences between groups in clinical trials setting. However, even in the case of statistically significant mean group effects, the intervention may not be effective for every participant in the study. A personalized approach to medical care is currently required (Damiano (2014)). It may also be the case that even for interventions deemed highly effective in CP, a range of individual responses may be observed, from a negative response to a strong positive effect. In order to better respond to the various needs of patients, it appears necessary to develop tools allowing a personalized approach based on a fine morphological analysis to propose a dedicated effective follow-up.

For this purpose, it is required to rely on dedicated analysis of the morphology of the ankle joint for each subject. Shape analysis aims at providing an automatic approach to compare shapes, to classify shapes by similarity or to detect morphological deformations. An analysis of the shape of the ankle bones could be used to identify and visualize the deformities, which could provide a better understanding of CP at the individual level as well as a more suitable rehabilitation program for those individuals (Kedem and Scher (2015)). Litterature reports the application of statistical shape model (SSM) on bone structures reconstruction, population-wise comparison and pathology detection, included but not limited to femur (Ebert et al. (2022); Asvadi et al. (2021); Boutillon et al. (2022); Shi et al. (2022)), pelvis (Vanden Berghe et al. (2017); Shi et al. (2022)), tibia (Shi et al. (2022); Schmutz et al. (2019)), fibula (Shi et al. (2022)) and scapula (Plessers et al. (2018); Boutillon et al. (2022); Salhi et al. (2020)).

However, a recent study (Goparaju et al. (2022)) has shown the importance of evaluation and validation of these tools in clinical applications. Specifically, this study compared three widely used state-of-the-art SSM tools, namely ShapeWorks (Cates et al. (2017)), Deformetrica (Durrleman et al. (2014); Bône et al. (2018)) and SPHARM-PDM (Styner et al. (2006)). The quantitative and qualitative results show that the SSM tools have different levels of consistency and different abilities to capture variability at the population level. What becomes apparent through this study is the need to compare results obtained using multiple shape analysis methods.

The objective of this work is therefore to provide comprehensive shape analysis of the ankle joint based on imaging studies. In order to propose a customized approach, we focus on tools to provide informative deformation maps at population level and also for each CP child based on shape analysis approaches. Morphometry, which is the study of the geometry of shapes, can be performed using voxel-based methods or surface-based approaches. In this work, we propose to investigate both types of approaches to provide the most comprehensive analysis possible at the patient level. Shape analysis relies on mapping (also called registration or matching) between subjects (or templates). Using a reference template, information about the individual shapes can be encoded in the deformation fields (Ashburner et al. (1998)). In such a context, personalized shape analysis is the study of the deformation fields for each subject.

In this article, we study the shape of the ankle joint of children with CP from high-resolution MRI data using two shape analysis approaches. More specifically, our contributions are three-folds: 1) a group analysis using two SSM methods, 2) an analysis of three ankle bones for a complete visualization of the joint, and 3) a subject analysis for a fine study of the deformation patterns for each child.

## 2 Materials and methods

### 2.1 Acquisition and preprocessing of clinical data

In this work, we focus on three bone shapes of the ankle joint: calcaneus, talus and tibia. Eleven TD children and nine children with CP with age ranged from 6 to 14 years old participated in this study which was approved by the regional ethics committee. The CP group includes seven males and two females and the TD group consists of seven males and four females with no history of pathology of the lower limbs. The demographic characteristics including age, weight, height and BMI of two groups are demonstrated in Table 1. The *T*-test is performed on these characteristics and no significant inter-group difference is noticed (*p* > 0.05). The details of demographic characteristics of each child included in the experiment are available in Supplementary Materials. All children were selected with no contraindications to MRI and with no history of lower limb musculo-skeletal injury or surgery in the past 6 months. MRI data were acquired in a single visit after parents signed informed consent forms.

**TABLE 1:** Demographic characteristics of the children included per group.

	Age (*years*)	Weight (*kg*)	Height (*cm*)	BMI (*kg*/*m* ^2^)
TD (mean ± std)	10.38 ± 1.95	38.89 ± 13.21	146.55 ± 14.75	17.93 ± 3.09
CP (mean ± std)	10.35 ± 2.39	32.7 ± 6.65	140.39 ± 11.01	16.54 ± 2.69
*p*-value	0.97	0.22	0.48	0.30

3D MRI data have been acquired using a 3T MR scanner (Achieva dStream, Philips Medical Systems, Best, Netherlands) with a resolution of 0.26 × 0.26 × 0.8* mm* ^*3*^ and resampled to 0.5 × 0.5 × 0.5 mm^3^ for the purpose of adaptation to clinic (T1-weighted gradient-echo, flip angle 10, matrix 576 × 576, FOV 150 mm × 150 mm, TR/TE 7.81/2.75 ms, mean acquisition duration: 424.32 s). Images of the ankle were taken on the CP group’s paretic lower limb, and on the non-dominant lower limb for the TD group. The acquisition protocol was detailed in Makki et al. (2019) and Garetier et al. (2020).

**FIGURE 2:**
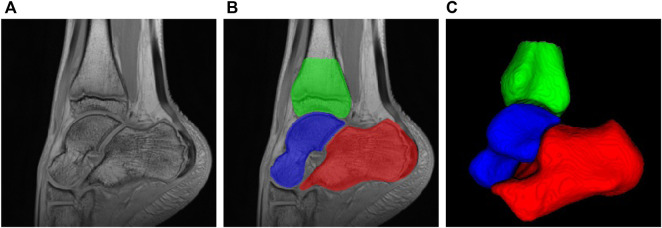
MRI of ankle joint and bone of interest: **(A)** Sagittal view of ankle static 3D MRI; **(B)** Segmentation of bone of interest: red: calcaneus, blue: talus, green: tibia; **(C)** 3D rendering of the three bones of interest.

In order to extract the shape of the 3 bones of interest, we make use of a semi-automatic segmentation approach. Age variability induces developmental variability of bones and cartilages. To overcome such shape variability, the considered regions of interest include bones and growth cartilages. First, 3 subjects at different ages (7, 10 and 12) are manually segmented by an expert. A registration-based label propagation method is then used to segment the subjects of the data sets, with manual correction if necessary. Newly segmented subjects are included at each propagation stage. Finally, smooth segmentation maps are computed by training a standard patch-based unet network Ronneberger et al. (2015) trained on the expert-based segmentation maps and manually corrected if required (see Figure 2).

The bone volume is an anatomical indice that related to muscle quality and thus the function, especially with cerebral palsy. The bone volume can be influenced by age, spasticity, muscle strength, and dorsiflexion range. In this study, we compare the volume of calcaneus and talus, including their growth cartilage, according to age. Figure 1 shows volume measurements of calcaneus and talus. These results, discussed in Section 3.1, tend to highlight morphological differences between the two groups, which we study next more precisely using two SSM approaches.

### 2.2 Surface-based shape analysis

Statistical Shape Modeling is a mathematical approach to quantify 3D shape variation. One approach for SSM is to analyze meshes computed for image segmentation. In this work, we rely on the surface-based SSM software called Shapeworks (Cates et al. (2017)) 1 that has been recently used for ankle joint analysis using weightbearing computed tomography (Krähenbühl et al. (2020); Lenz et al. (2021)). Recent benchmarking study has shown the potential of Shapeworks with respect to other surface-based SSM (Goparaju et al. (2022)). ShapeWorks is a groupwise particle-based shape modeling method that does not rely on surface parameterizations. Shapeworks handles each surface mesh as a set of particles that describe the surface geometry. Such a particle-based representation avoids many of the problems inherent in parametric representations (i.e. limitation to specific topologies for instance). Shapeworks takes as input binary segmentation of each bone of every subjects. Correspondences between surfaces (relying on particles) are estimated using signed distance images. Procrustes analysis is used to remove scaling (i.e. size) from the shape modeling analysis. Mean shapes are generated for CP and TD groups and deformation fields between the two groups are used to study the shape differences.

### 2.3 Voxel-based shape analysis

**FIGURE 3:**
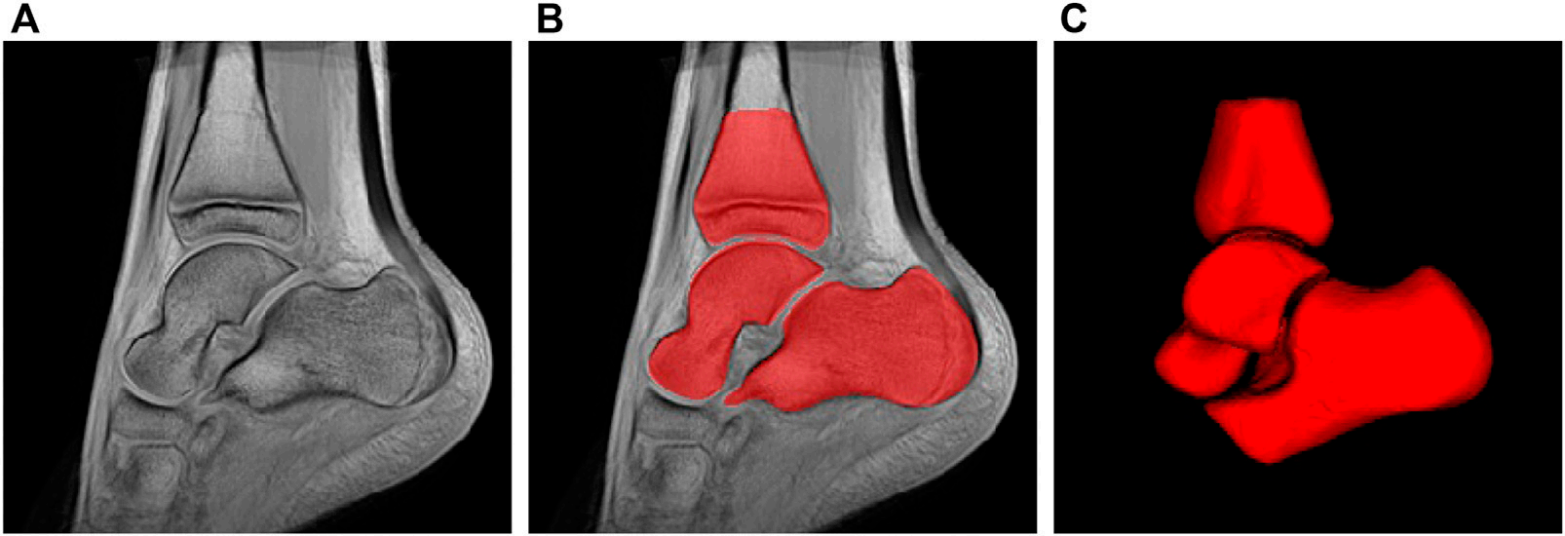
Estimated ankle atlas of TD population using the voxel-based approach: **(A)** sagittal view, **(B)** segmentation, **(C)** 3D rendering.

For the voxel-based approach, we make use of an image registration-based framework to compute a mean image model of the TD population. To deal with the high variability in shape and appearance of the bones, we compute the TD template using a group-wise diffeomorphic algorithm (Avants and Gee (2004)) by not only considering image intensity but also bone segmentation maps using signed distance maps. Such a multivariate approach ensures realistic template estimation (see Figure 3) with sharp details of cortical bones and cartilage. Then, for each subject of the dataset, deformation fields are estimated by non-linear multivariate registration onto the mean TD template previously computed. The template estimation and the patient-to-template registration stages are performed with Advanced Normalization Tools (ANTs)^2^.

## 3 Results

### 3.1 Global scale analysis–volumetric quantification

In a first step, we perform a bone volumetric quantification of calcaneus and talus, from a global perspective, to provide a global view of bone morphological difference caused by equinus. The mean calcaneus volume of TD and CP group are 48,172.9* mm* ^*3*^ and 41,001.6* mm* ^3^, and for talus are 29,404.1* mm* ^3^ and 25,138.3* mm* ^*3*^. Both calcaneus and talus are decreased by 15% in CP group compared to TD group. The gap between 2 groups is increased with age, as presented in Figure 1.

### 3.2 Analysis at group level

**FIGURE 4:**
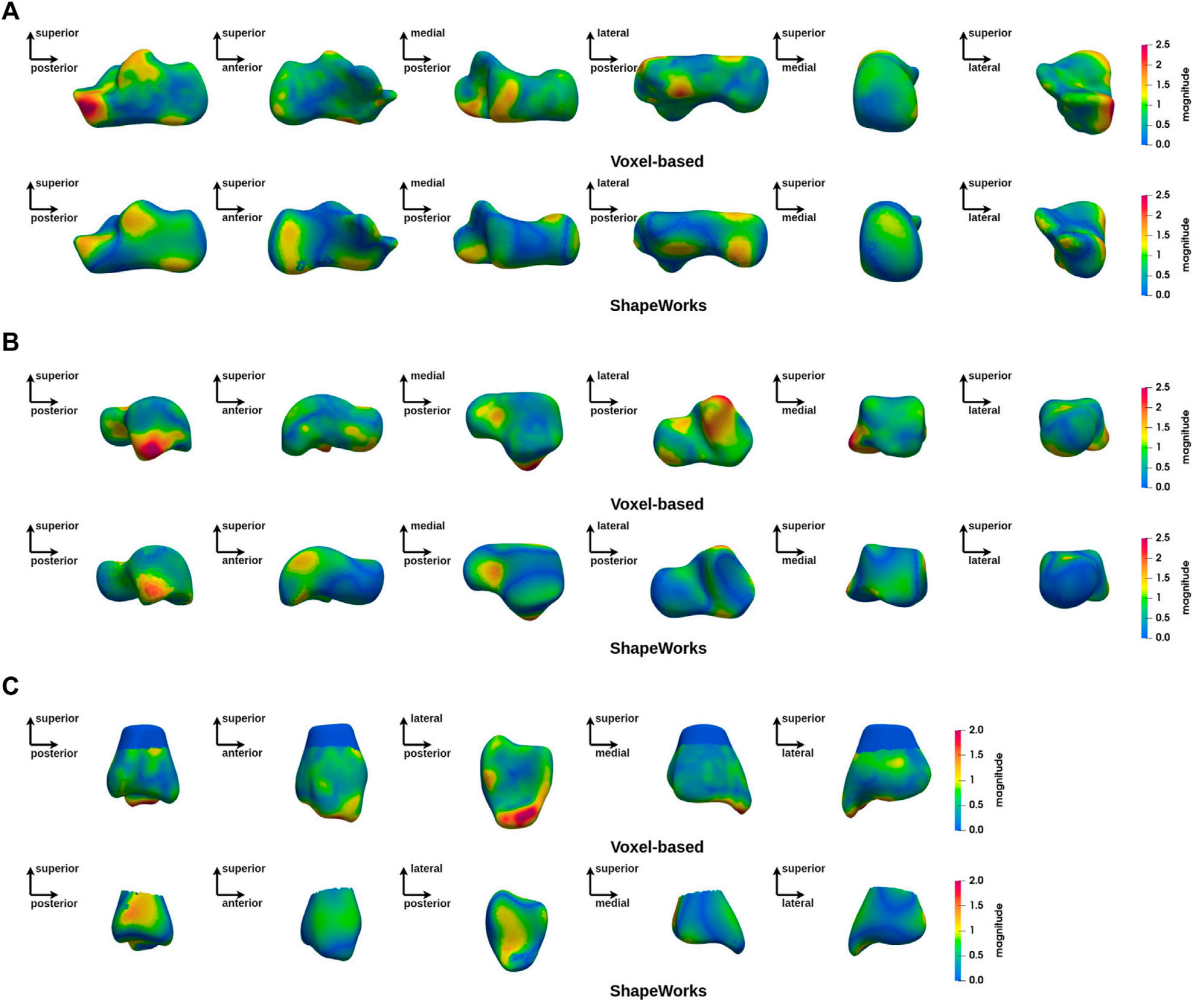
Magnitude of deformation fields at group level (CP vs. TD) using ShapeWorks and voxel-based methods: **(A)** calcaneus, **(B)** talus, **(C)** tibia.

Our first objective is to provide a comparison between the two groups of interest (CP and TD), with the voxel-based and particle-based methods. Figure 4 shows the magnitude of the deformation fields for the 3 bones of interest from the CP group toward the TD group. These deformations correspond to the average shape variations between the two populations. This figure indicates that the two SSM methods studied in this work provide similar results regarding the main deformation regions of talus and calcaneus. These results tend to show that both SSM methods capture the same patterns of shape variation at the group level for these two bones.

**FIGURE 5:**
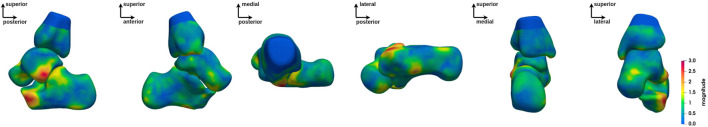
Magnitude of deformation fields at group level (CP vs. TD) using the voxel-based analysis.

Figure 5 shows a group-wide deformation zone in the lateral process of the talus and a deformation zone on the anterolateral aspect of the calcaneus. These two areas are anatomically opposite each other. A zone of deformity is also seen on the inferolateral aspect of the tibial malleolus.

### 3.3 Analysis at subject level

**FIGURE 6:**
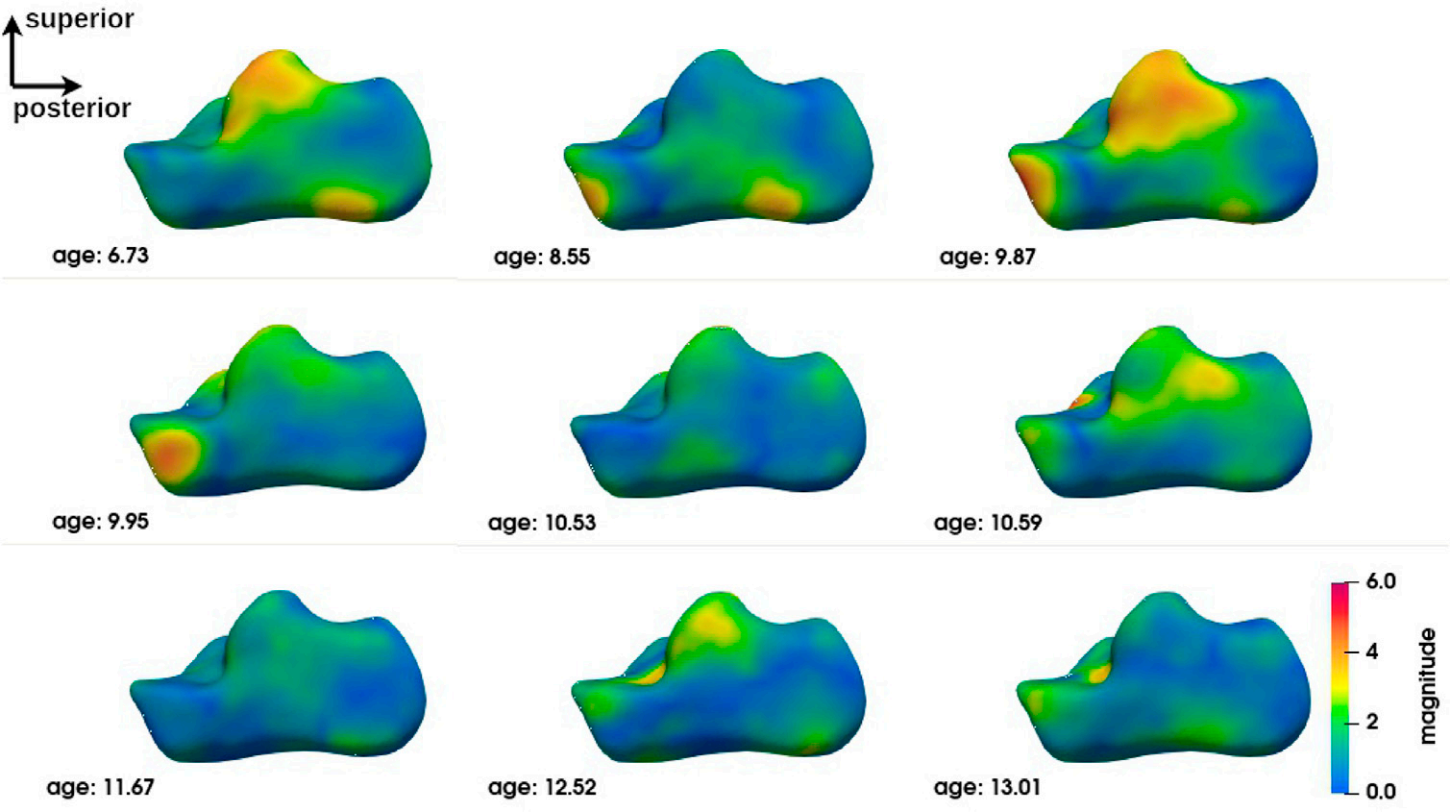
Subject-level shape analysis of calcaneus in lateral view of CP population.

**FIGURE 7:**
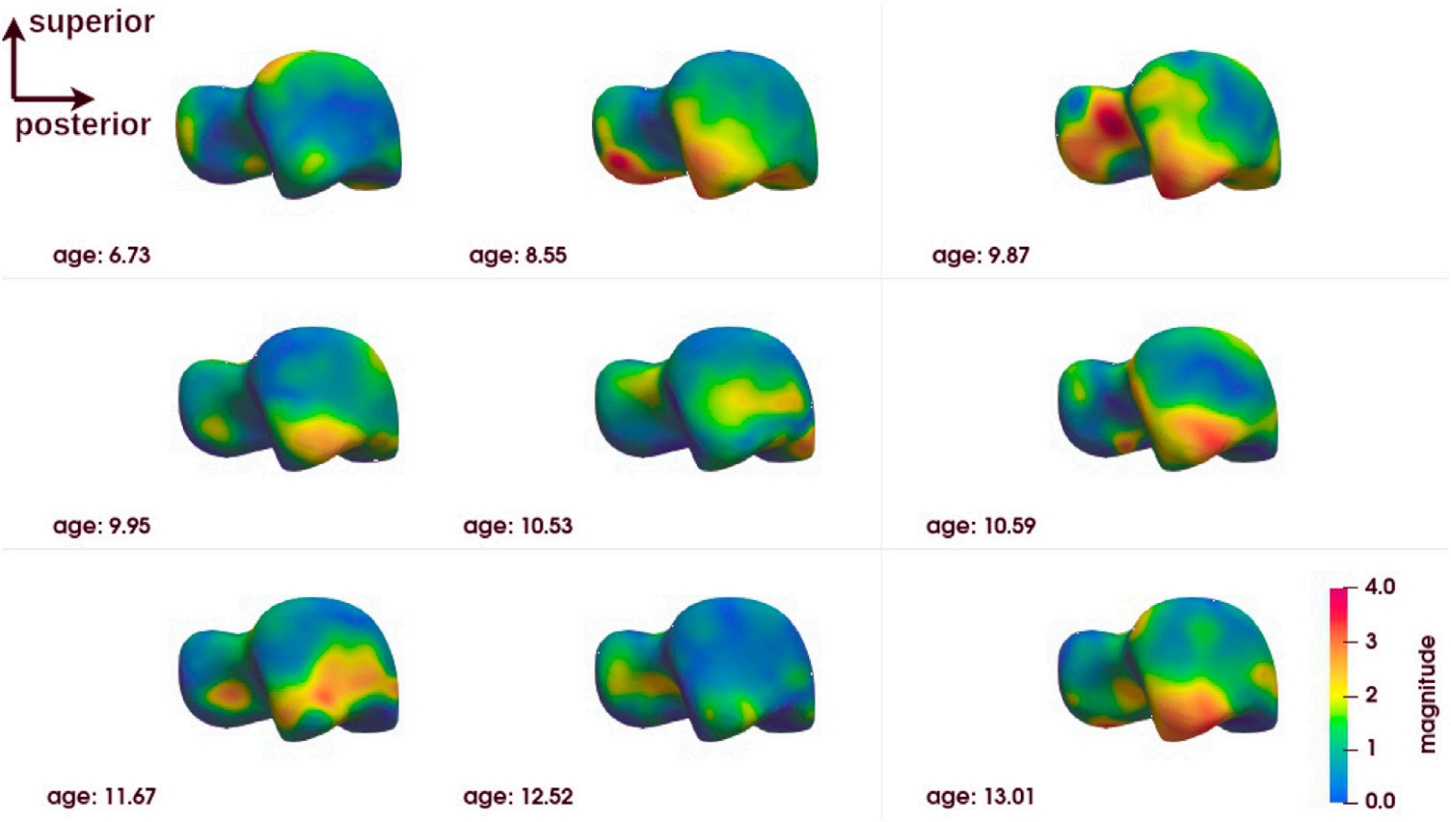
Subject-level shape analysis of talus in lateral view of CP population.

**FIGURE 8:**
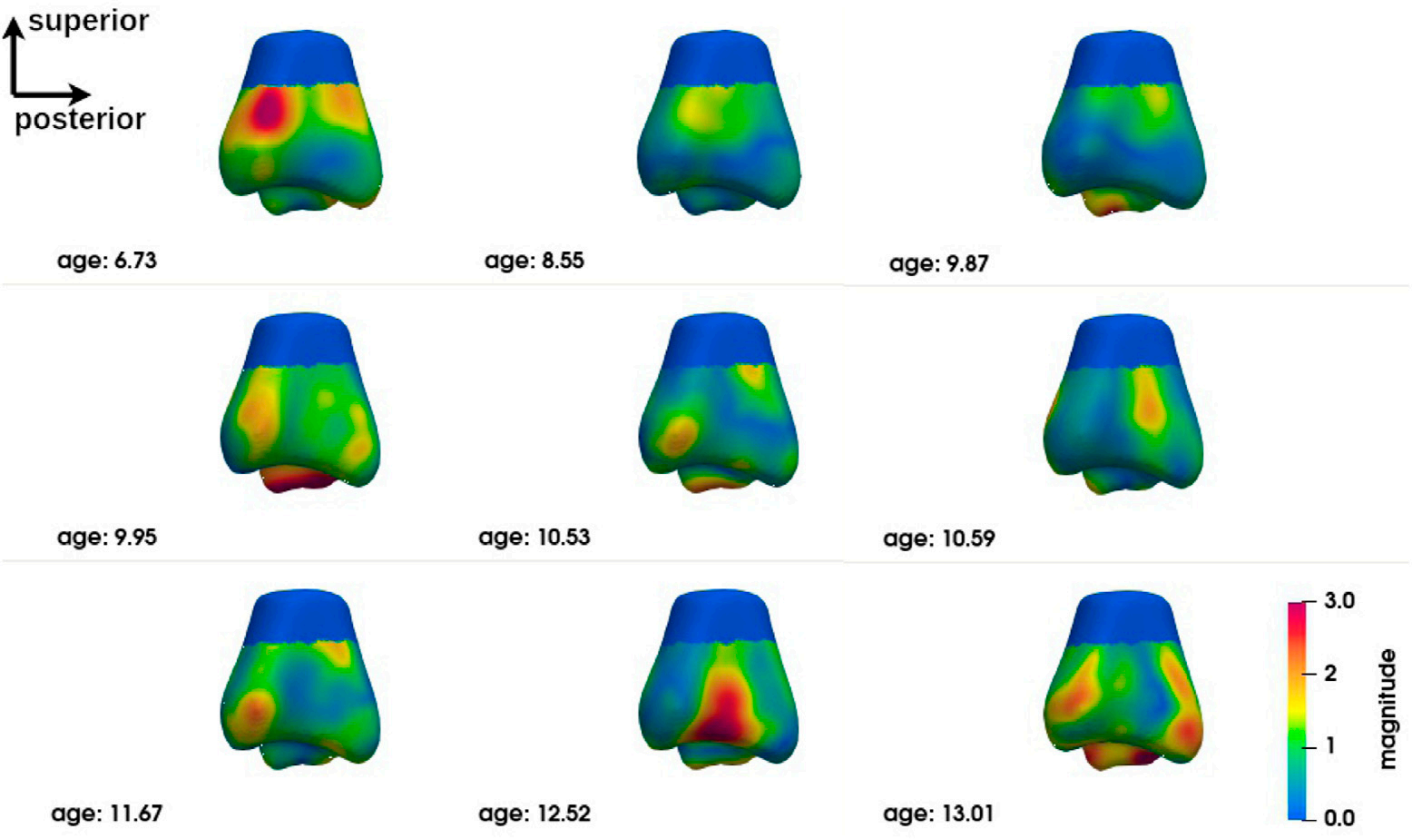
Subject-level shape analysis of tibia in lateral view of CP population.

The group-level analysis shows shape differences on some regions of the bones of interest. However, the group-level analysis only provides average deformation patterns, without allowing a personalized analysis for each child. An analysis at the individual level is necessary to propose a more personalized approach. Figures 6–8 show the magnitude of the deformation fields between each CP subject (ordered by age) and the TD atlas using the voxel-based method for respectively, the calcaneus, the talus and the tibia in one view. The other views can be found in Supplementary Materials. The main deformation patterns revealed by the group analysis may be observed in some subjects, but not necessarily in all subjects. Age does not seem to be related to the observed deformation patterns.

## 4 Discussion

Motivated by the observed volume difference of calcaneus and talus between TDs and CPs, this study is aimed to investigate the ankle joint bone morphology relevant to fixed equinus caused by cerebral palsy. To understand population-wise pathological change and to adjust patient-adapted rehabilitation program, the morphological analysis was performed on both on population level and individual level. To this end, we make use of two SSM methods. Such an analysis provides the possibility to analyse the morphological properties and detect large deformations than can be caused by pathology (Frondelius et al. (2022); Brown et al. (2017)).

### 4.1 Cross-approach comparison

In Gao et al. (2014) and Goparaju et al. (2022), authors described different levels of consistency between SSM tools and stressed the importance of validating these tools in medical applications. Different SSM methods can lead to results with large variations. Under these conditions, a comparison between several shape analysis methods is necessary.

In this study, we adopt a surface-based approach and a voxel-based approach. Both methods capture similar group-level deformation regions for talus and calcaneus. Indeed, both methods reveal deformations of the anterior lateral edge of the calcaneus, which is located near the cuboid facet and the anterior facet of the subtalar joint, as well as the posterior-lateral edge of the posterior facet of the subtalar joint and the lateral process of the calcaneal tuberosity. In the talus, the area showing obvious deformity in both methods is the lateral process, as well as the neck of the talus. Compared to the calcaneus and the talus, the results for the tibia show less similarity between the methods. However, deformities of the infero-lateral part of the tibial malleolus are evident, which in relation to the deformities found in the calcaneus and talus can be explained by the valgus deformity of the hindfoot frequently found in children with CP and equinus gait. This deformity, exacerbated by weight-bearing, causes dislocation of the hindfoot with malalignement of the ankle bones, increasing local mechanical stress (Otjen et al. (2020)). The increase in stress is consistent with the deformities seen in the lateral-anterior border of the calcaneus, the lateral process of the talus and the tibial malleolus.

However, differences still exist between two methods. In calcaneus, the calcaneus tuberosity and its medial process have large deformation magnitude in surface-based approach, while this change is not reported by voxel-based approach. In talus, the lateral side of posterior facet is reported by voxel-based approach but not by mesh-based approach. In tibia, the fibula notch region obtained with mesh-based method has a magnitude of 1 mm while approximately 0.5 mm in voxel-based analysis.

### 4.2 Subject-level analysis

The subject-level analysis reveals the deformation pattern of each CP subject. As presented in Section 3.3, the subject-level analysis may correspond to the group analysis, but not necessarily.

Interestingly, the talus appears to be the bone with the least variation in inter-subject deformation. At the same time, it appears to be the bone of the ankle complex with the most deformation. This may be related to its anatomical-physiological characteristics (absence of muscle insertion, poor vascularisation) and its anatomical position at the crossroads of the mechanical constraints of the talus and tibia (Dufour (2015)). Just as it is more sensitive to osteochondral lesions in sportsmen and women (Barbier et al. (2021)), it seems to be more sensitive to deformation in CP individuals with equinus gait. This suggests that the evolution of the shape of the talus should be monitored more closely.

Despite this trend, no precise deformation pattern is observed for any of the three bones of the ankle complex. Although individual #3 (age 9.87) appears to be closest to the deformation pattern described above at the group level and some individuals are close to it in some deformations, alternative patterns are found for each individual. This does not mean that these individuals do not have deformities that could be explained by their loaded foot position or their gait. Further exploration is required to establish these relationships. The presence of major deformities at the individual level means that individualised diagnostic imaging assessment, in conjunction with gait analysis, should be encouraged in order to provide specific and personalized treatment.

### 4.3 Limitations and perspectives

This study offers exciting new information concerning the bone morphology of children with cerebral palsy and equinus. However, it is necessary to continue such a study in order to confirm these results. Firstly, this study is performed on a limited-size dataset due to the scarcity of pediatric datasets. Although the similar deformation pattern is revealed from our dataset with different tools, it would be better to confirm our results by increasing the number of subjects included. The inter-group difference (in terms of age and sex) of the subjects who participated in the study requires caution in interpreting the results. The results must therefore be analysed in relation to the study population and must be confirmed on the basis of a larger data set. Secondly, as already highlighted in Gao et al. (2014) and Goparaju et al. (2022), this work confirms the need to analyze the results obtained with SSM methods with care and a future line of research must be the understanding of the potential variability of the results obtained by SSM tools. It is essential to provide simple and efficient tools for shape analysis in a personalized context.

## Data Availability

The raw data supporting the conclusion of this article will be made available by the authors, without undue reservation.

## Footnotes

1
https://sciinstitute.github.io/ShapeWorks.

2
http://stnava.github.io/ANTs/.
