# Supplementary material for: Comprehensive personalized ankle joint shape analysis of children with cerebral palsy from pediatric MRI
Source: Front Bioeng Biotechnol. 2022 Nov 25;10:1059129. doi: 10.3389/fbioe.2022.1059129 (PMC9732549; doi:10.3389/fbioe.2022.1059129)
Supplement: Supplementary file 1 [file DataSheet1.zip › supplementary/supplementary_v3.pdf]

Supplementary Material

1 DEMOGRAPHIC CHARACTERISTICS OF SUBJECTS

	Gender	Age $(years)$	Weight (kg)	Height (cm)	BMI (kg/m^2)
TD01	M	8.07	28.3	135.0	15.53
TD02	F	10.59	38.1	148.0	17.39
TD03	F	10.61	49.6	149.0	22.34
TD04	M	12.00	52.2	156.0	21.45
TD05	F	12.51	52.2	162.0	19.89
TD06	F	7.53	18.4	118.0	13.21
TD07	M	13.17	46.6	157.5	18.79
TD08	M	12.31	33.2	149.0	14.95
TD09	M	12.59	56.9	162	21.68
TD10	M	7.44	27.2	129	16.35
TD11	M	7.00	25.1	126.5	15.69

Table S1. Demographic characteristics of subjects in TD group.

	Gender	Age $(years)$	Weight (kg)	Height (cm)	BMI (kg/m^2)
CP01	M	9.95	26.8	134.5	14.81
CP02	F	12.52	37	150.0	16.44
CP03	M	13.01	39.8	160.0	15.55
CP04	M	9.87	25.4	135.0	13.94
CP05	M	10.59	30.9	138.5	16.11
CP06	M	11.67	40.6	143.0	19.85
CP07	F	10.53	31.4	147.0	14.53
CP08	M	6.73	23.3	122.5	15.53
CP09	M	8.55	39.1	133.0	22.10

Table S2. Demographic characteristics of subjects in CP group.

2 SUPPLEMENTARY VIEWS OF SUBJECT-LEVEL ANALYSIS

To complete the visualization of subject-level analysis, the other views except the lateral visualization angel are presented here. Fig. S1 to S5 are the anterior, inferior, medial, posterior and superior views of calcaneus. The talus of all these five views is represented by S6 to S10. Fig. S11 to S14 are of tibia without the proximal end, since this study focuses on the distal end of tibia which contributes to ankle joint function. Same as in lateral view, the subject-level analysis shows the similarity to the group analysis, as well as the variation in different CP subjects. Group analysis cannot accurately represent the deformation patterns of all subjects. A patient-specific treatment program is necessary.

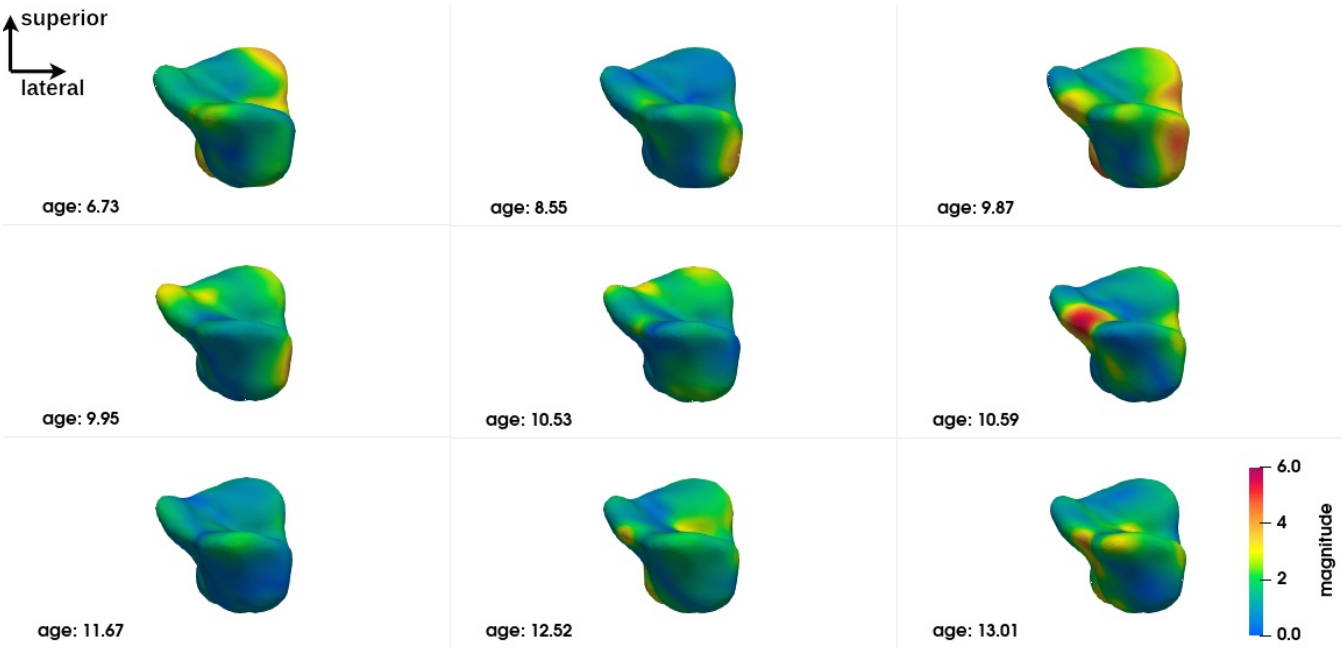

Figure S1. Subject-level shape analysis of calcaneus in anterior view

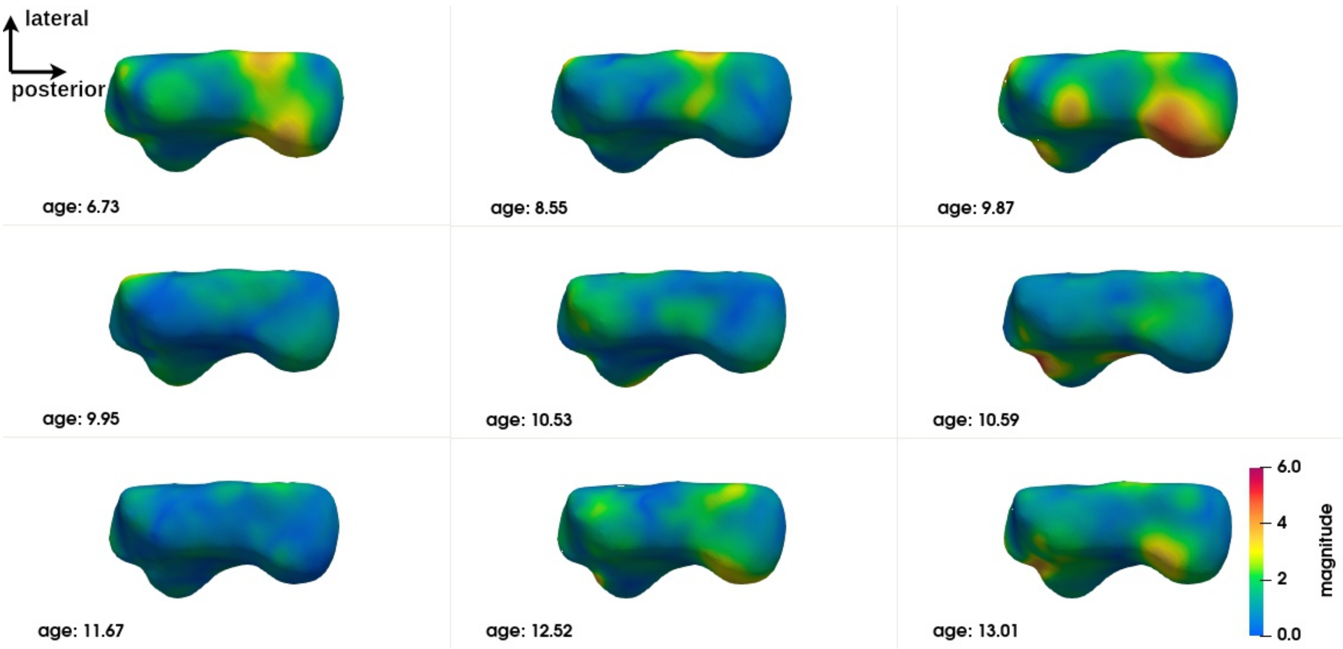

Figure S2. Subject-level shape analysis of calcaneus in inferior view

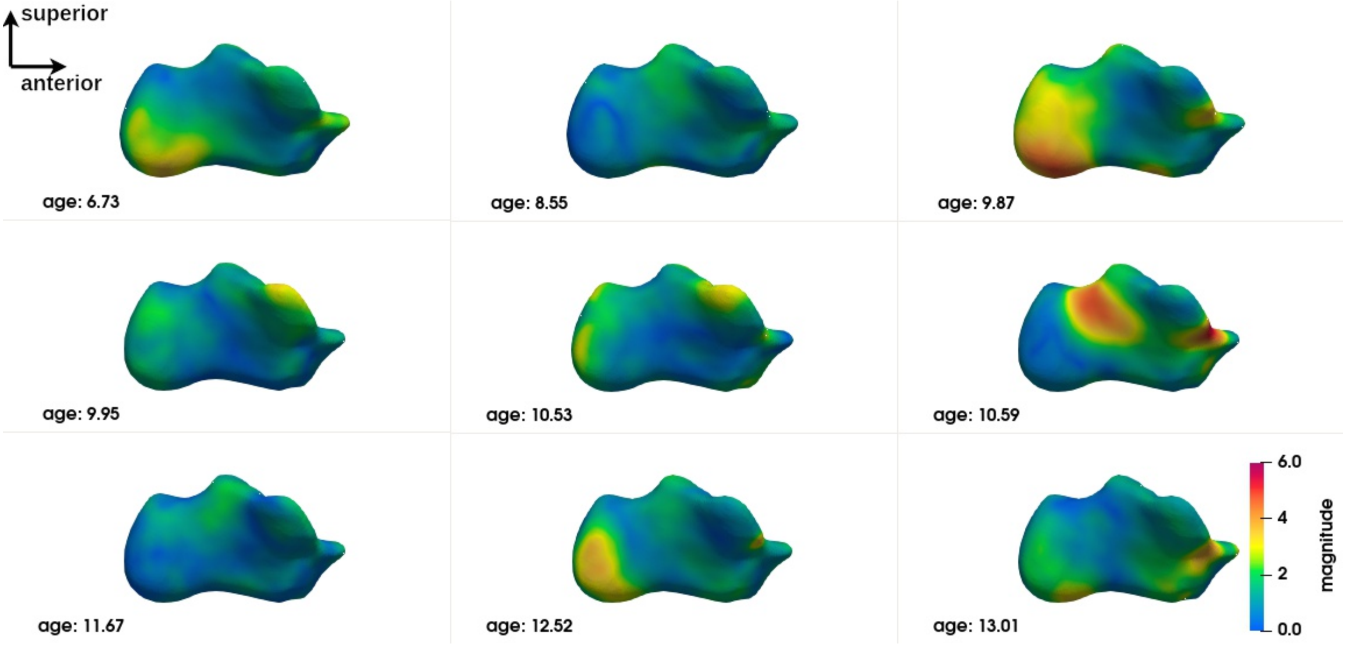

Figure S3. Subject-level shape analysis of calcaneus in medial view

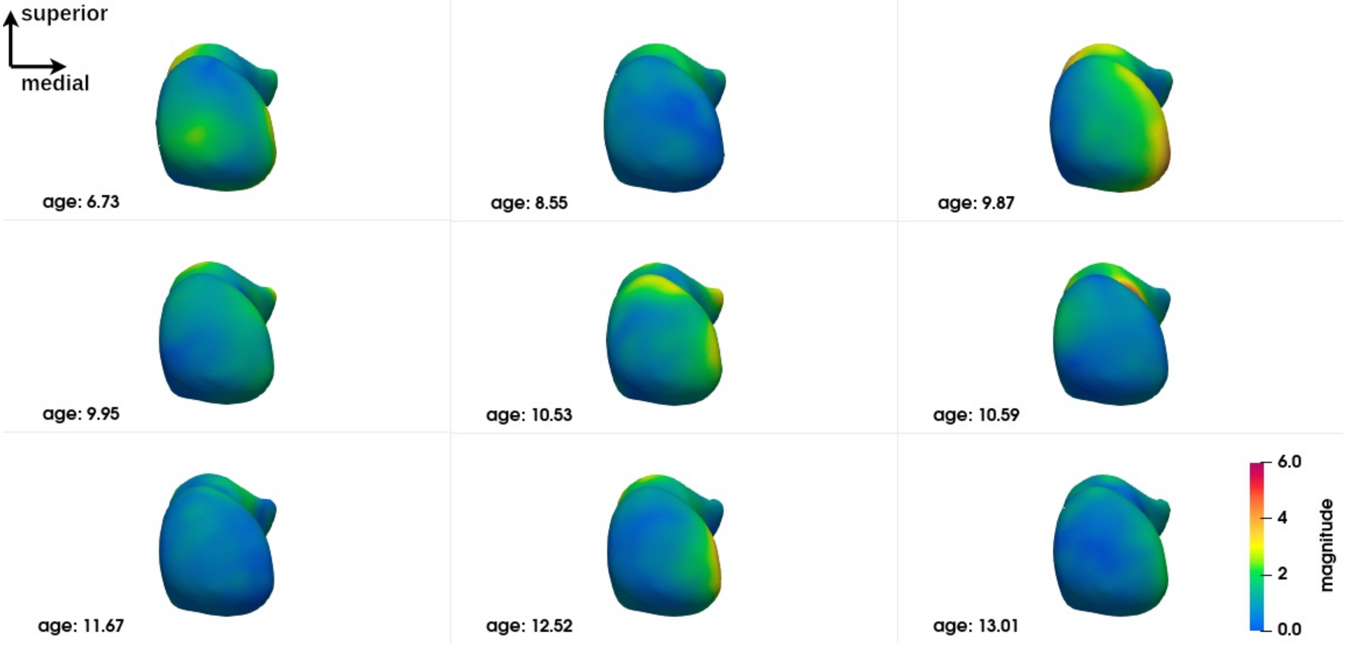

Figure S4. Subject-level shape analysis of calcaneus in posterior view

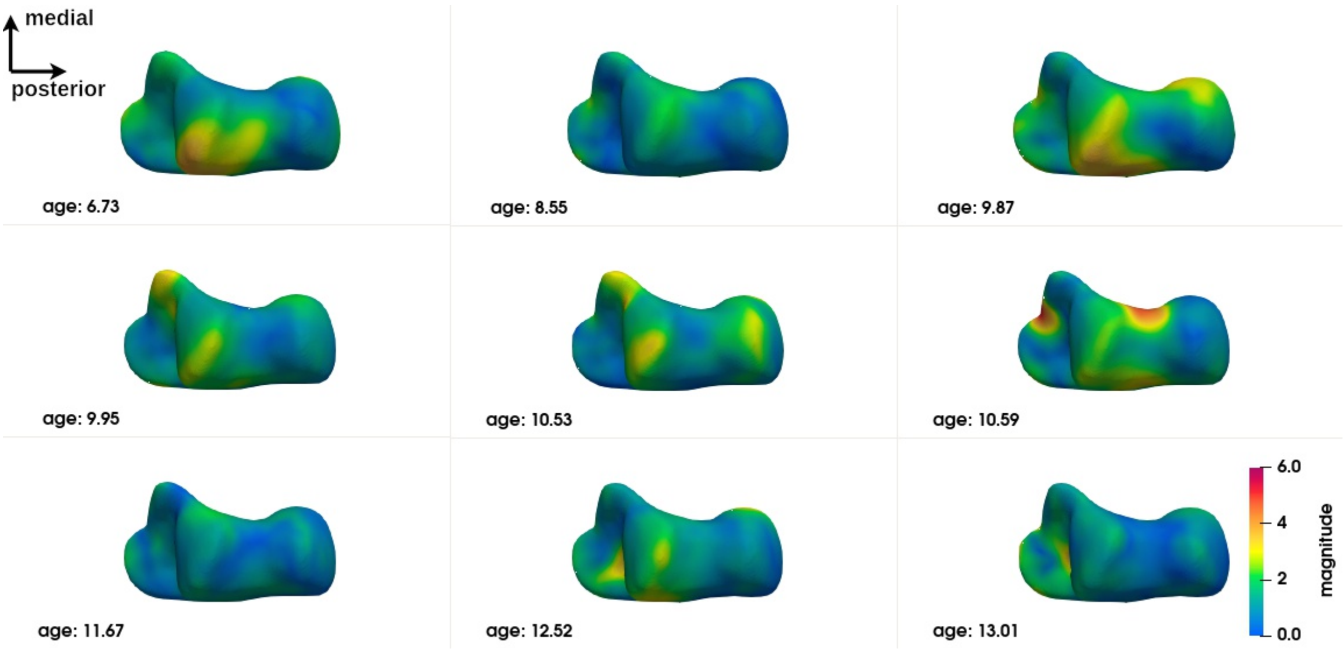

Figure S5. Subject-level shape analysis of calcaneus in superior view

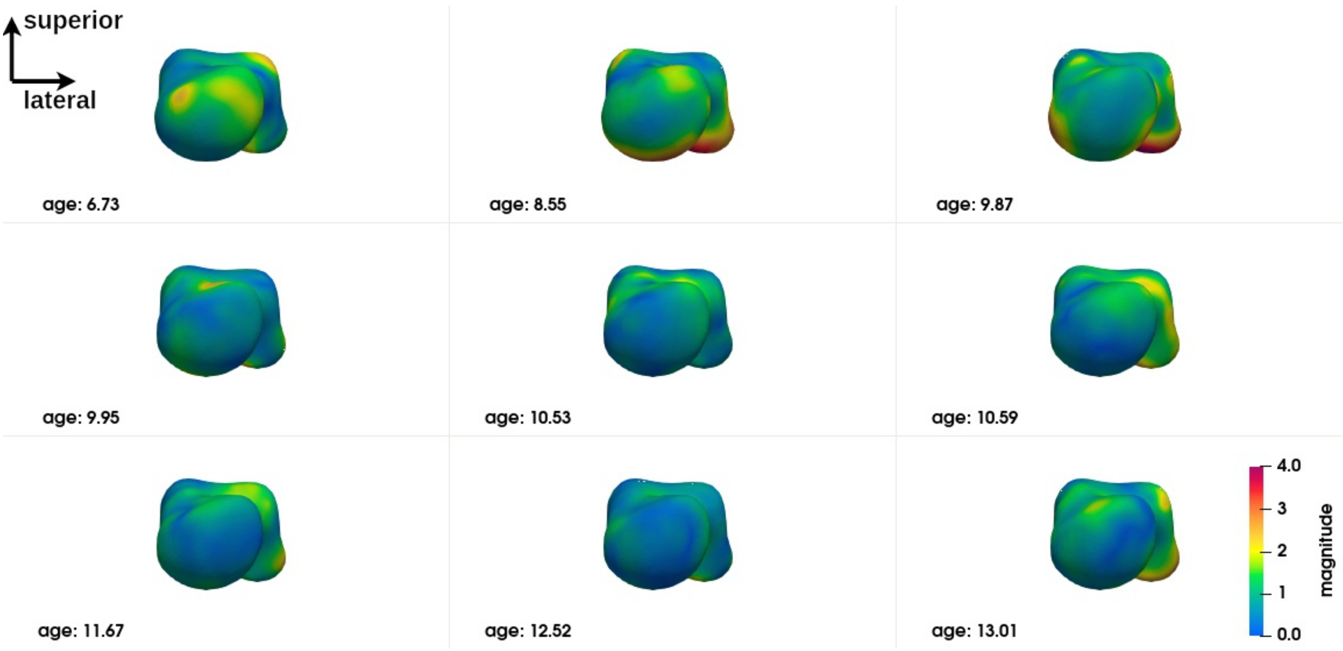

Figure S6. Subject-level shape analysis of talus in anterior view

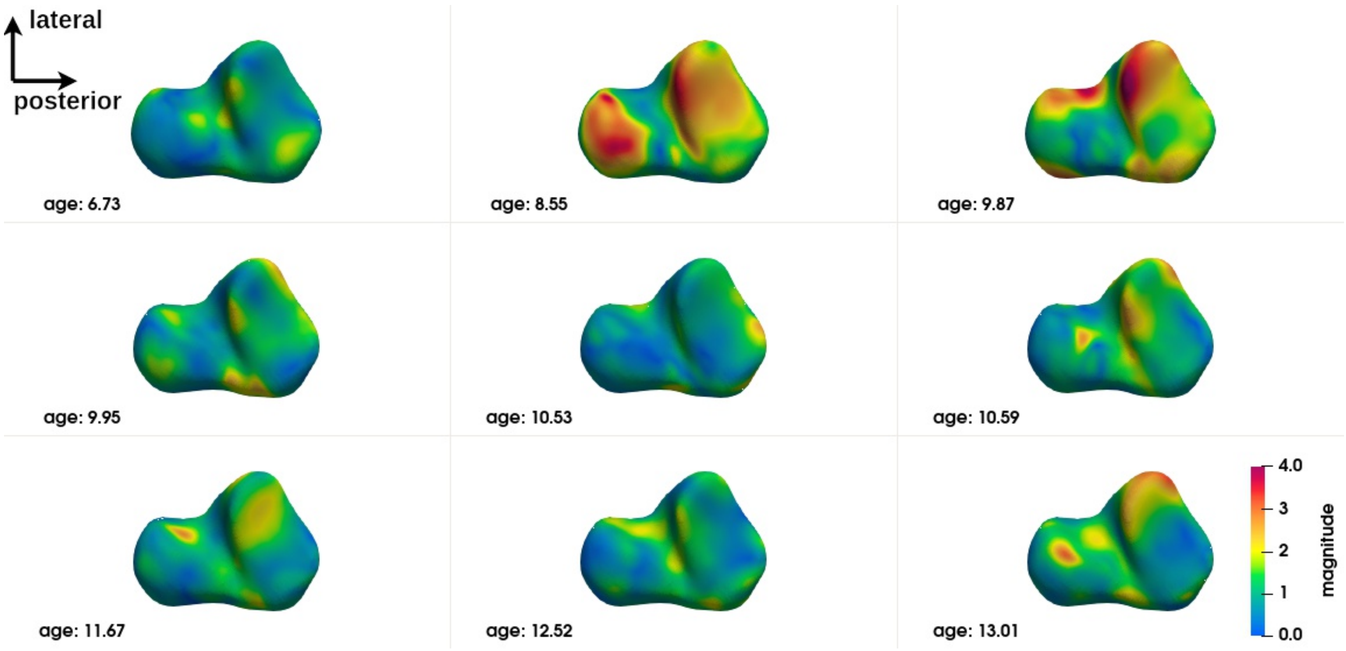

Figure S7. Subject-level shape analysis of talus in inferior view

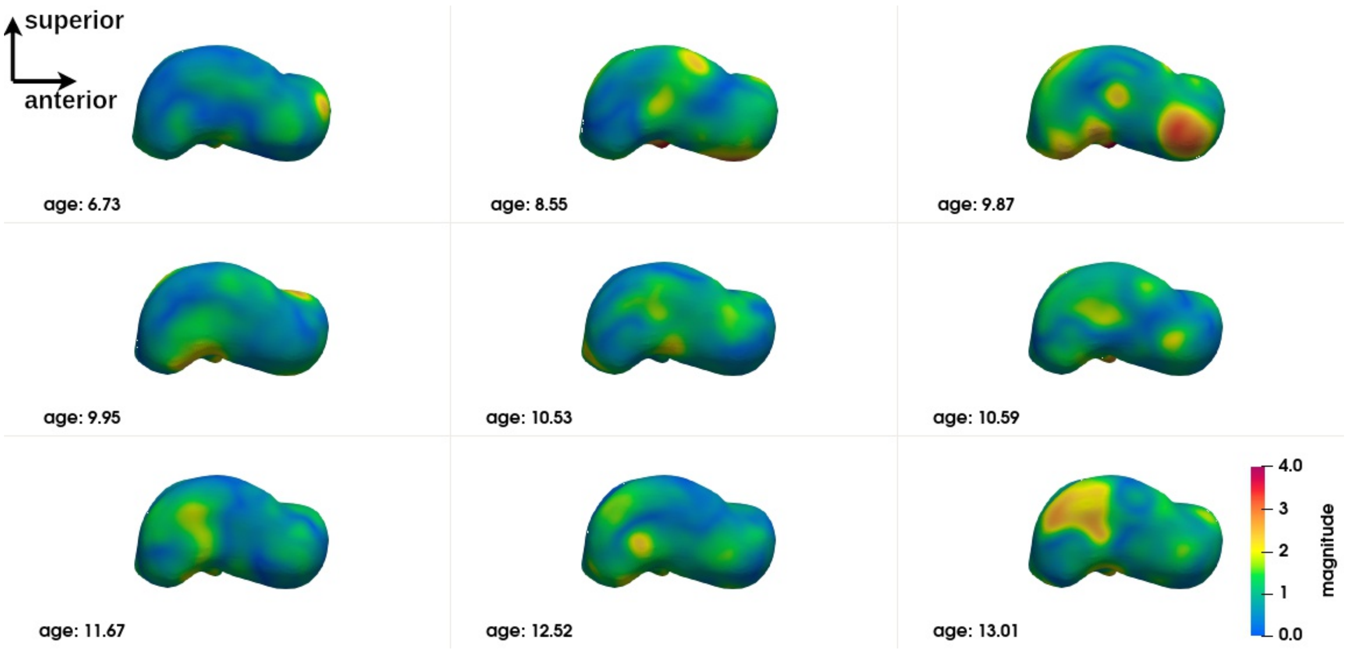

Figure S8. Subject-level shape analysis of talus in medial view

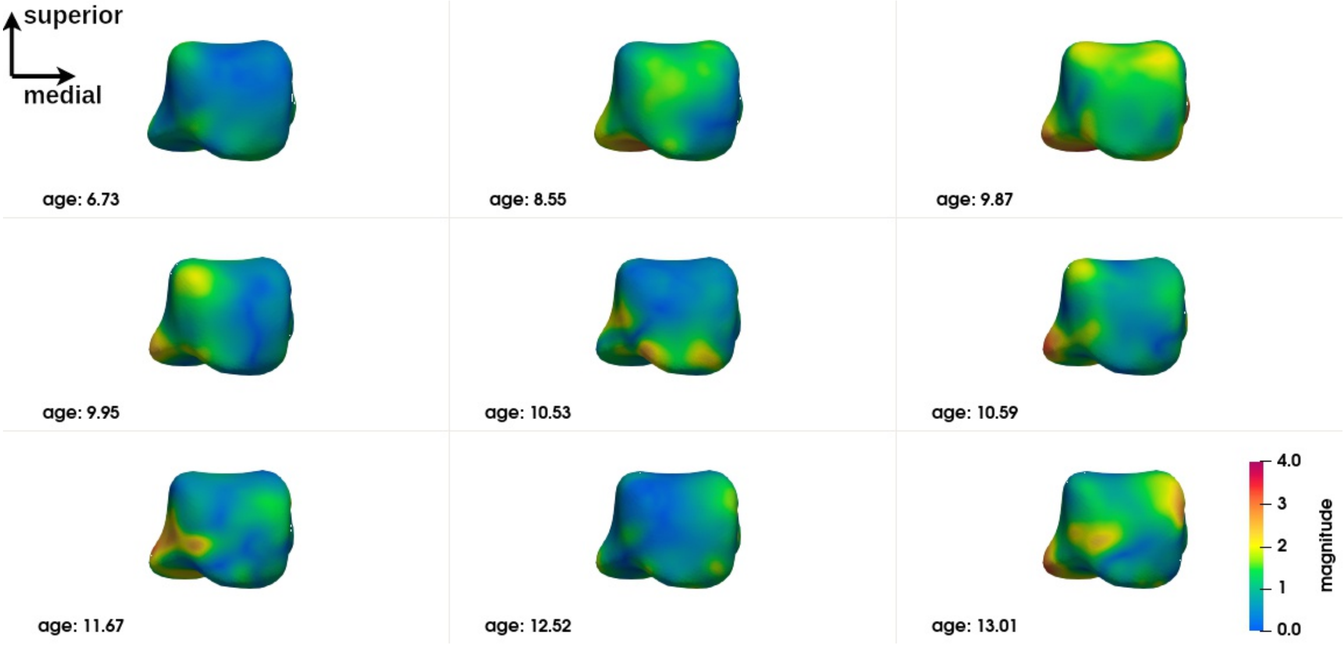

Figure S9. Subject-level shape analysis of talus in posterior view

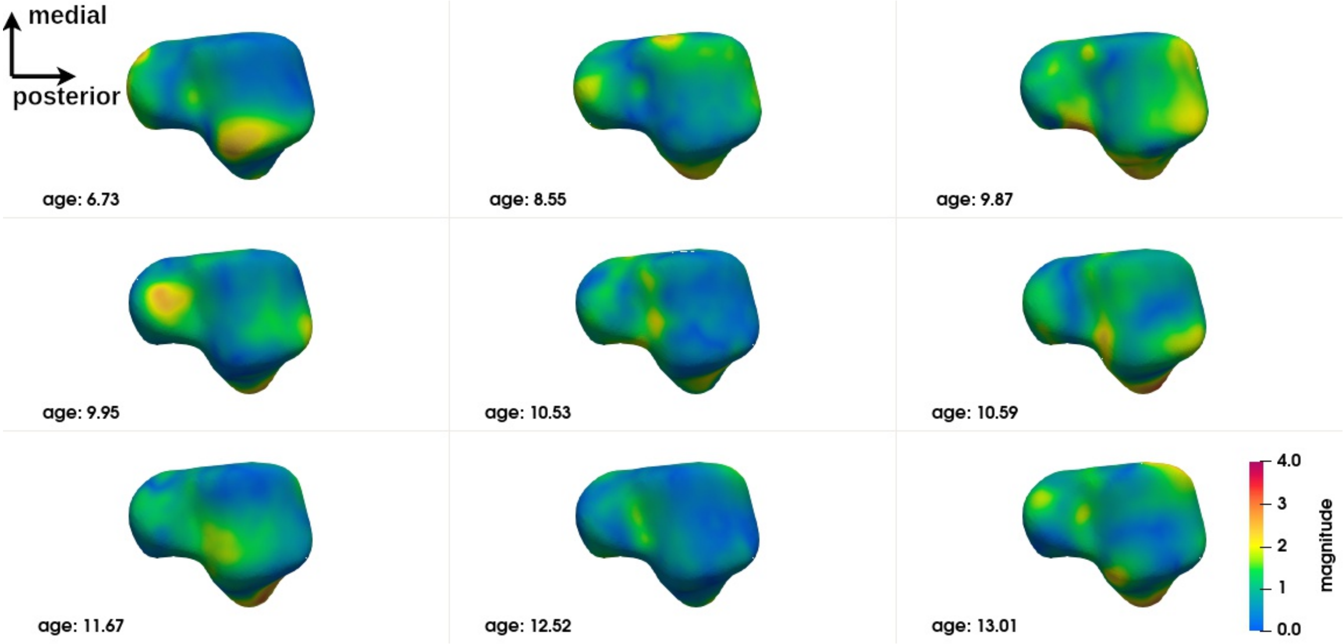

Figure S10. Subject-level shape analysis of talus in superior view

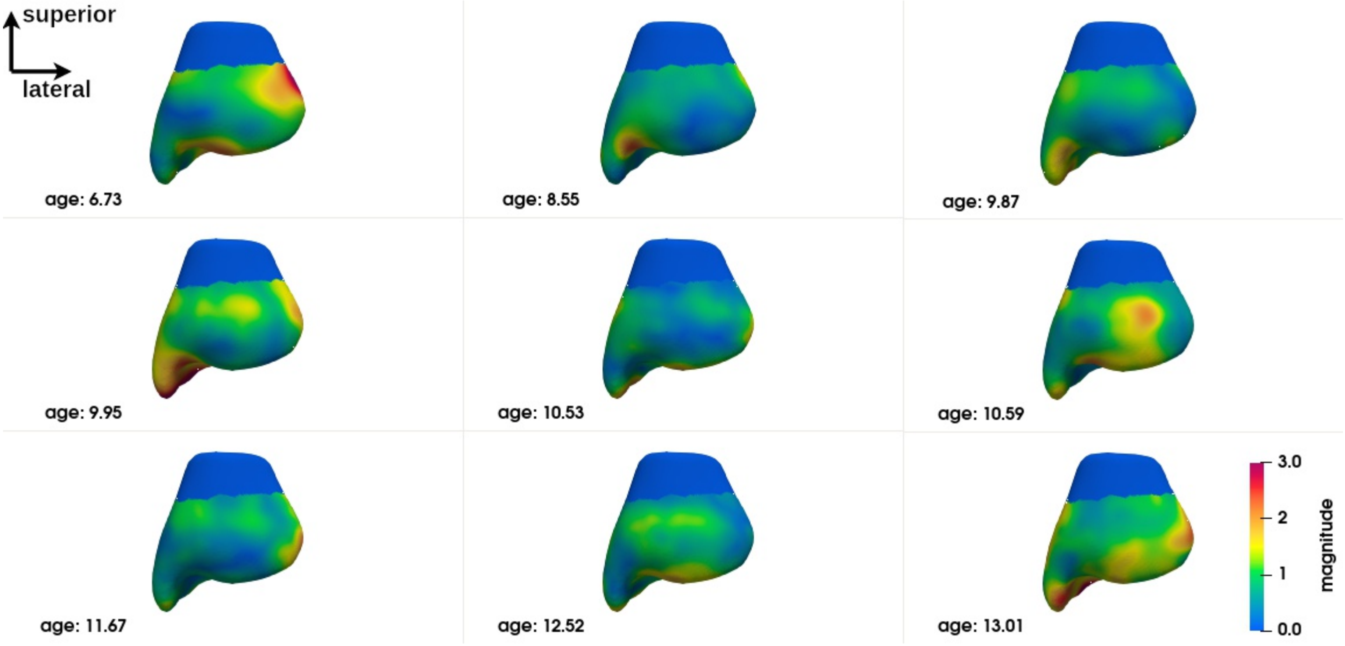

Figure S11. Subject-level shape analysis of tibia in anterior view

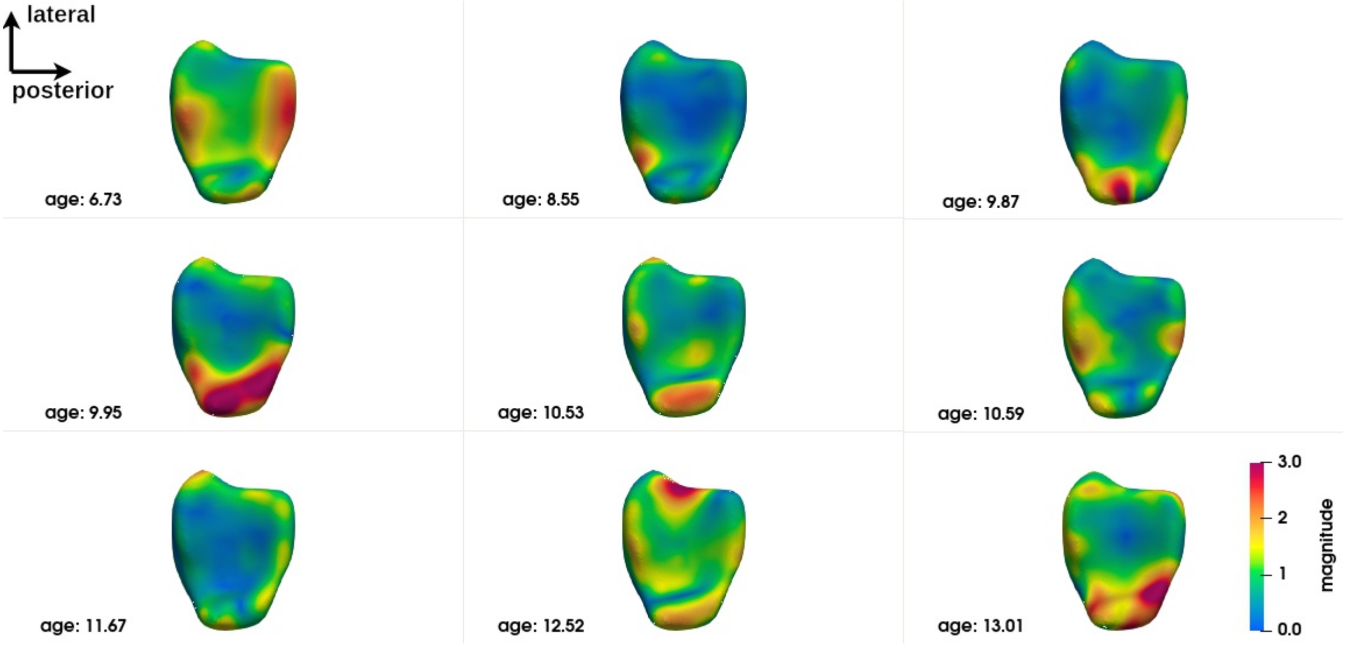

Figure S12. Subject-level shape analysis of tibia in inferior view

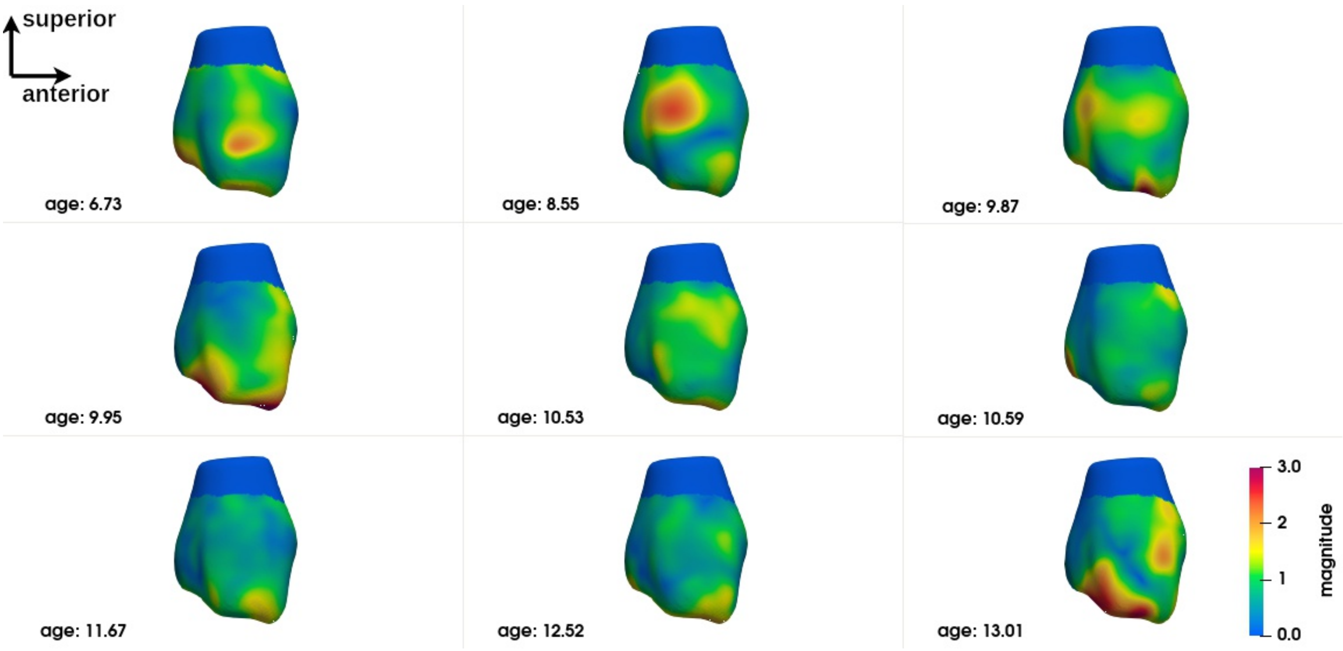

Figure S13. Subject-level shape analysis of tibia in medial view

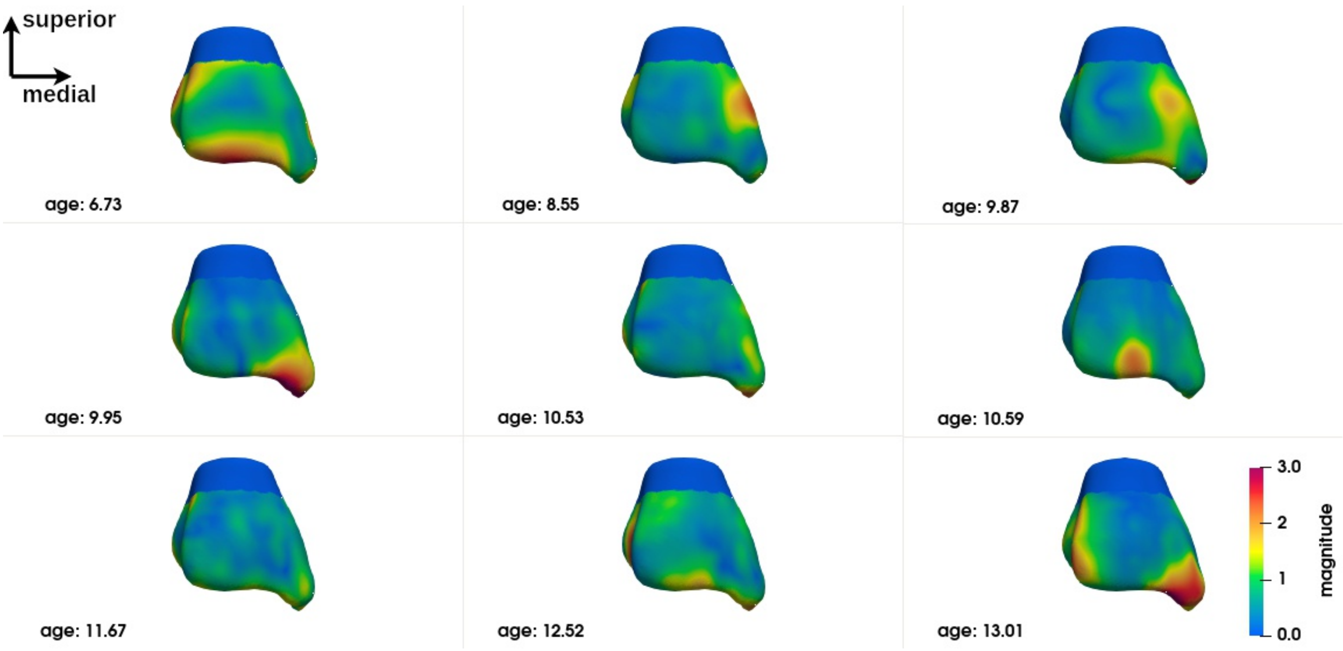

Figure S14. Subject-level shape analysis of tibia in posterior view
