# Supplementary figures and images for: Comprehensive personalized ankle joint shape analysis of children with cerebral palsy from pediatric MRI
Source: Front Bioeng Biotechnol. 2022 Nov 25;10:1059129. doi: 10.3389/fbioe.2022.1059129 (PMC9732549; doi:10.3389/fbioe.2022.1059129)

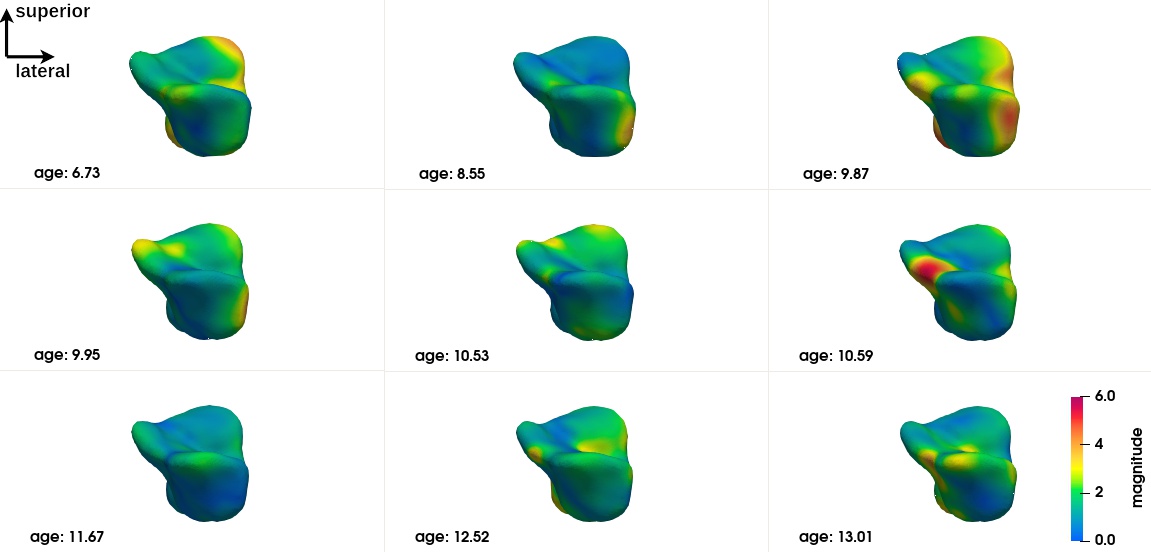

Supplement: Supplementary file 1 [file DataSheet1.zip › supplementary/Image 1.JPEG]

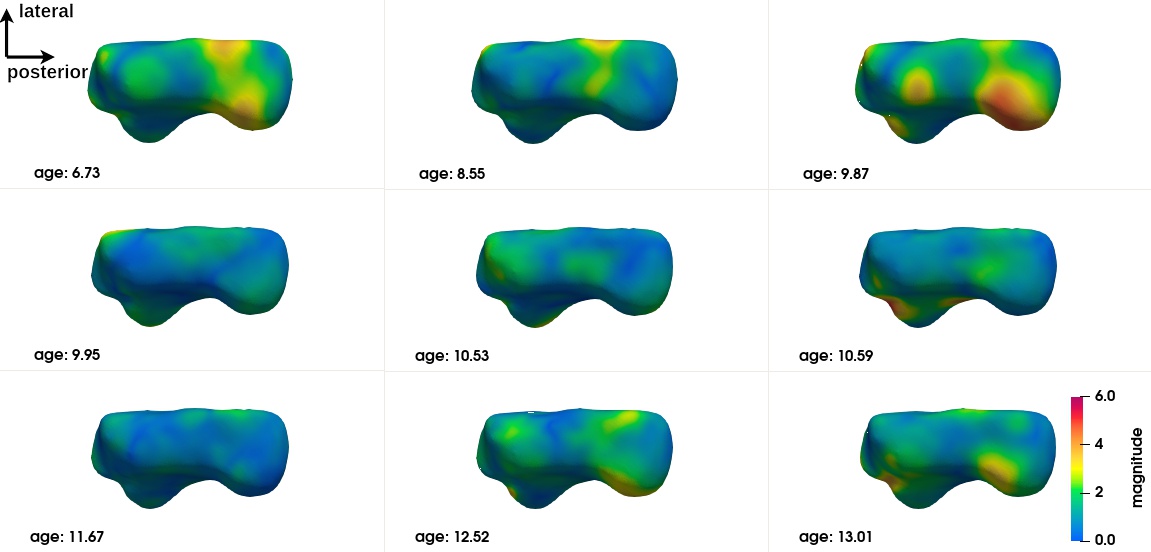

Supplement: Supplementary file 1 [file DataSheet1.zip › supplementary/Image 2.JPEG]

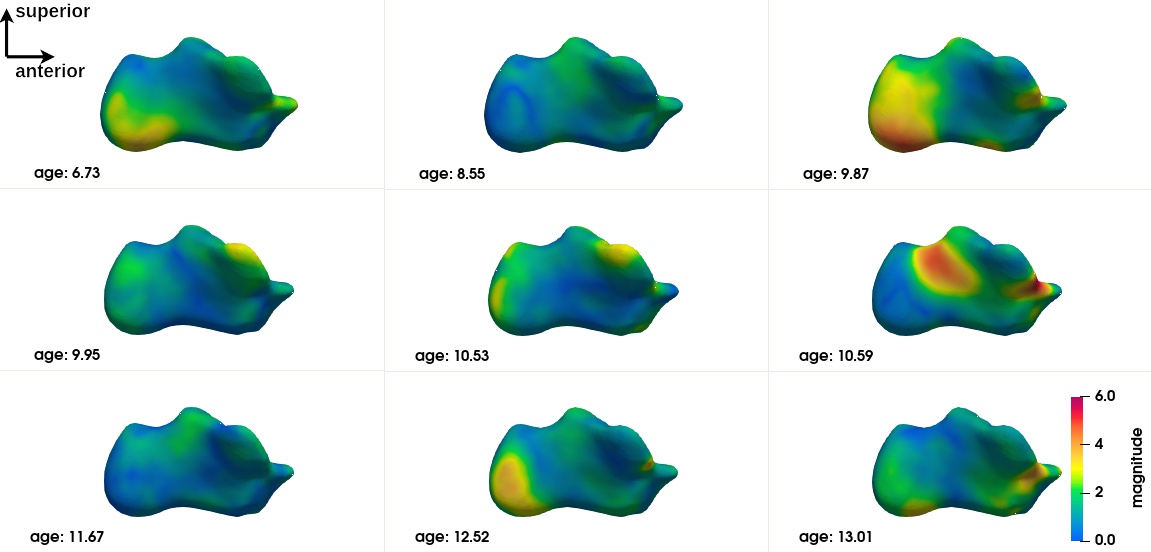

Supplement: Supplementary file 1 [file DataSheet1.zip › supplementary/Image 3.JPEG]

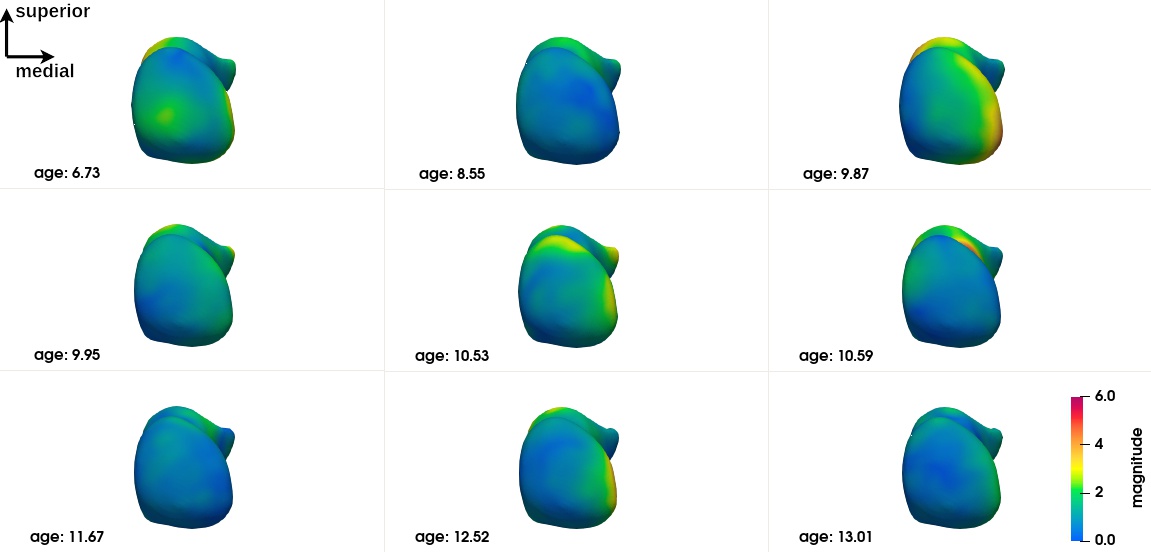

Supplement: Supplementary file 1 [file DataSheet1.zip › supplementary/Image 4.JPEG]

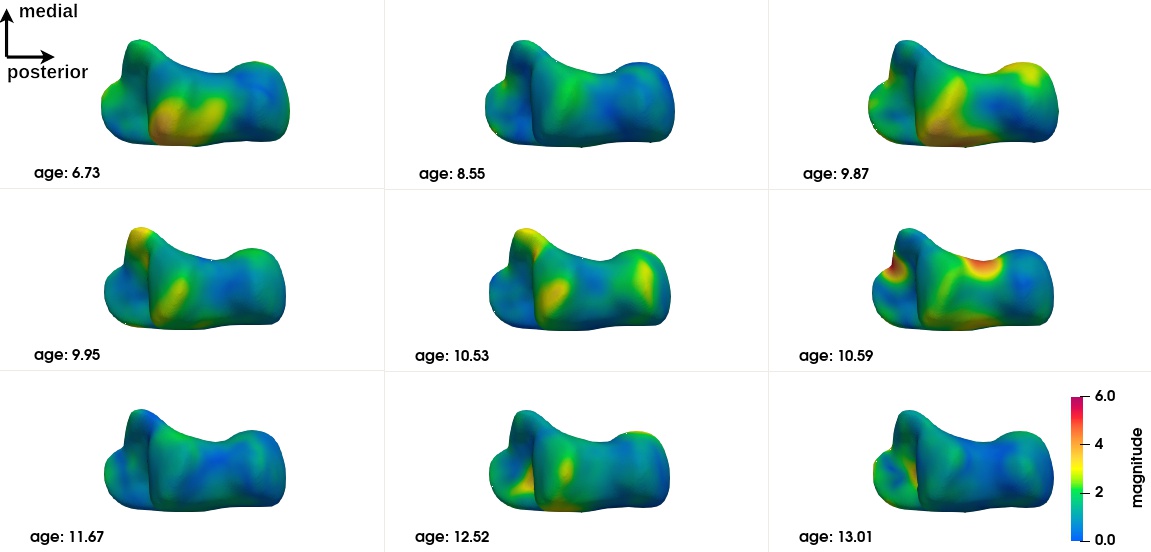

Supplement: Supplementary file 1 [file DataSheet1.zip › supplementary/Image 5.JPEG]

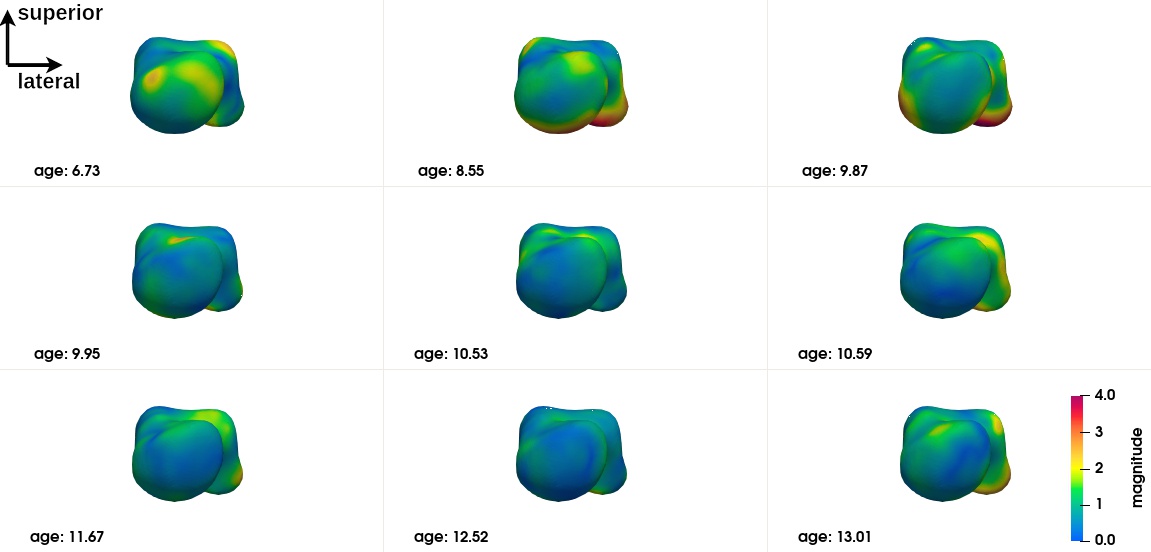

Supplement: Supplementary file 1 [file DataSheet1.zip › supplementary/Image 6.JPEG]

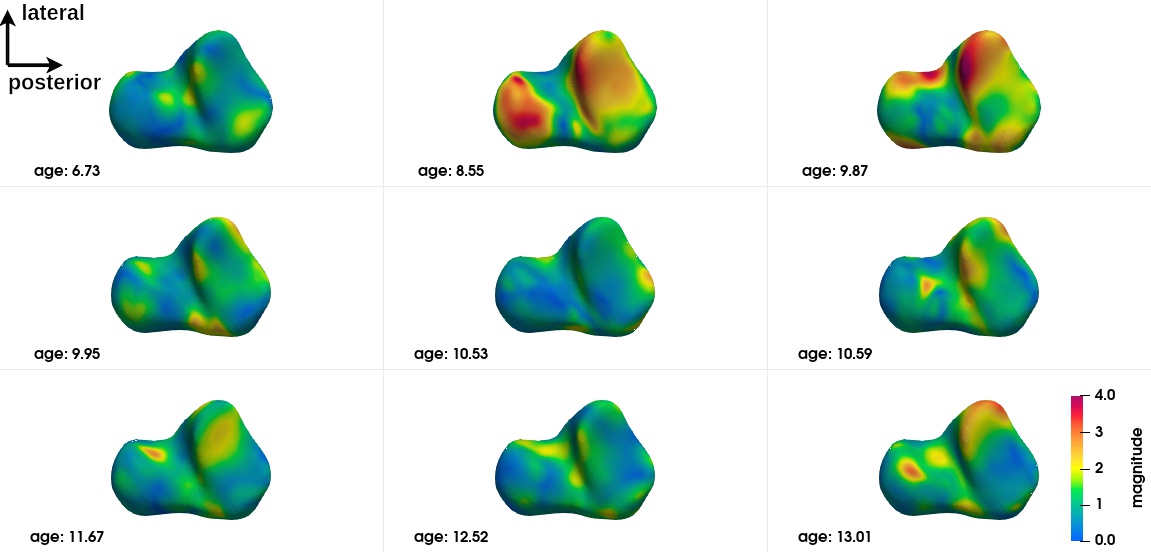

Supplement: Supplementary file 1 [file DataSheet1.zip › supplementary/Image 7.JPEG]

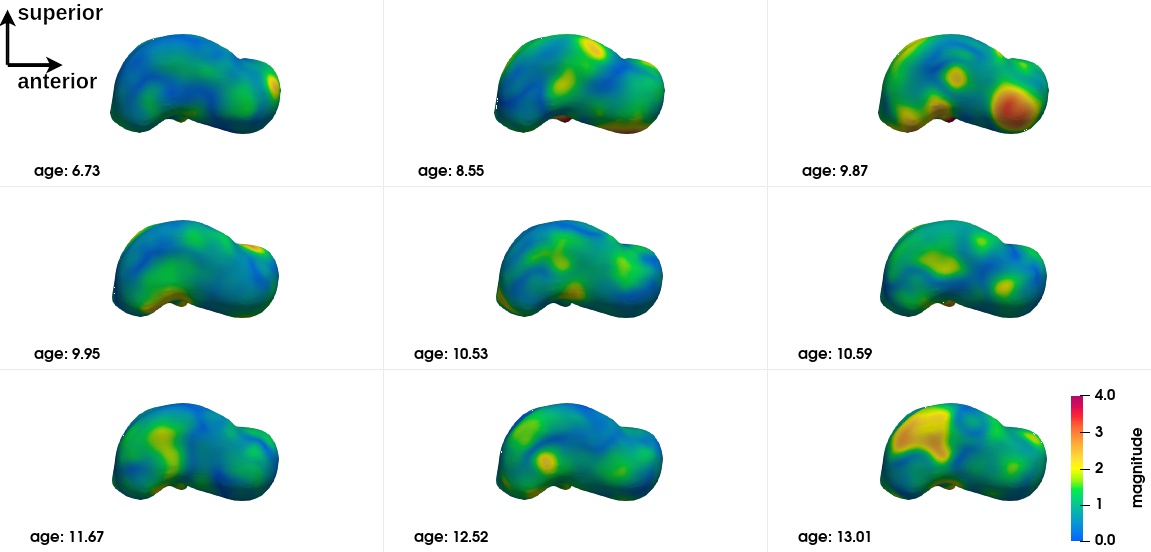

Supplement: Supplementary file 1 [file DataSheet1.zip › supplementary/Image 8.JPEG]

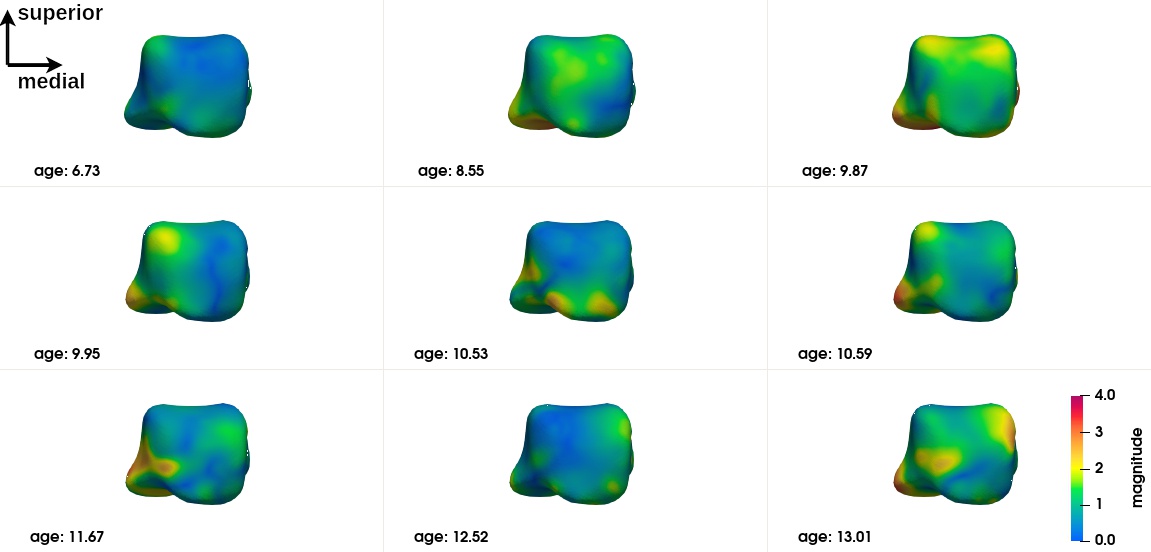

Supplement: Supplementary file 1 [file DataSheet1.zip › supplementary/Image 9.JPEG]

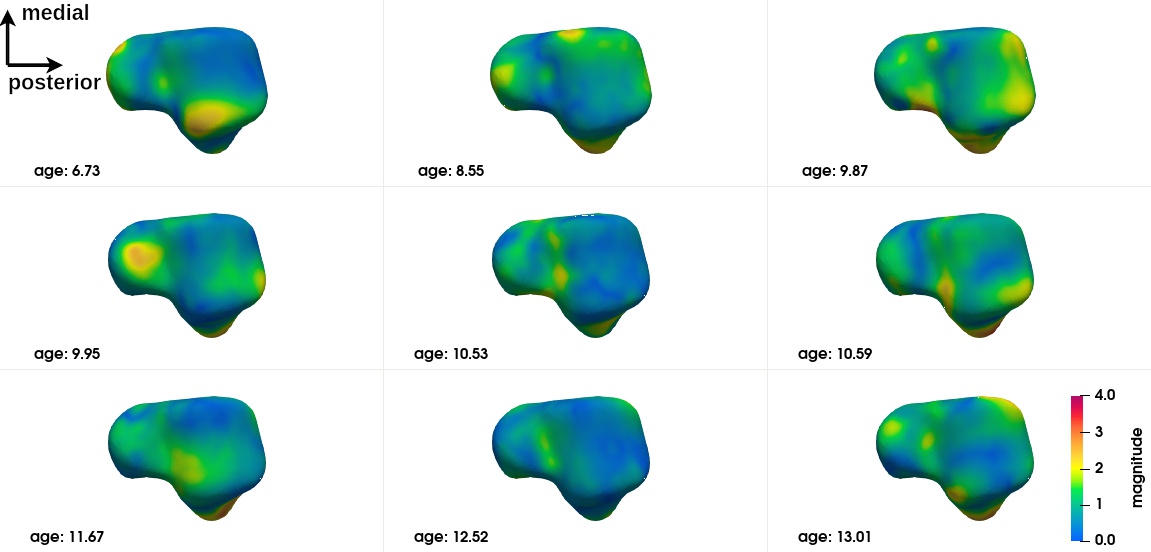

Supplement: Supplementary file 1 [file DataSheet1.zip › supplementary/Image 10.JPEG]

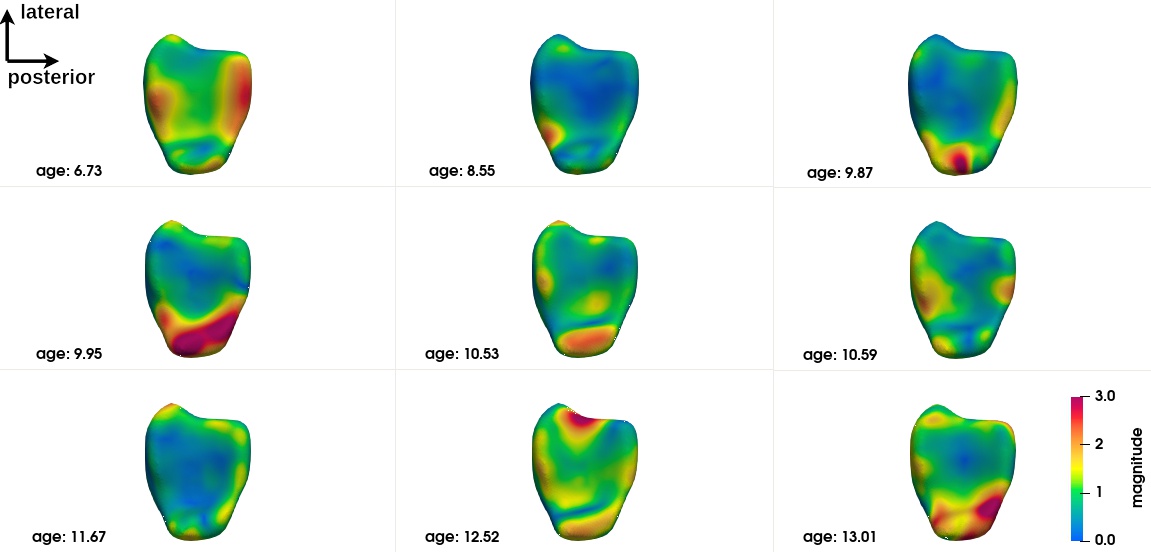

Supplement: Supplementary file 1 [file DataSheet1.zip › supplementary/Image 12.JPEG]

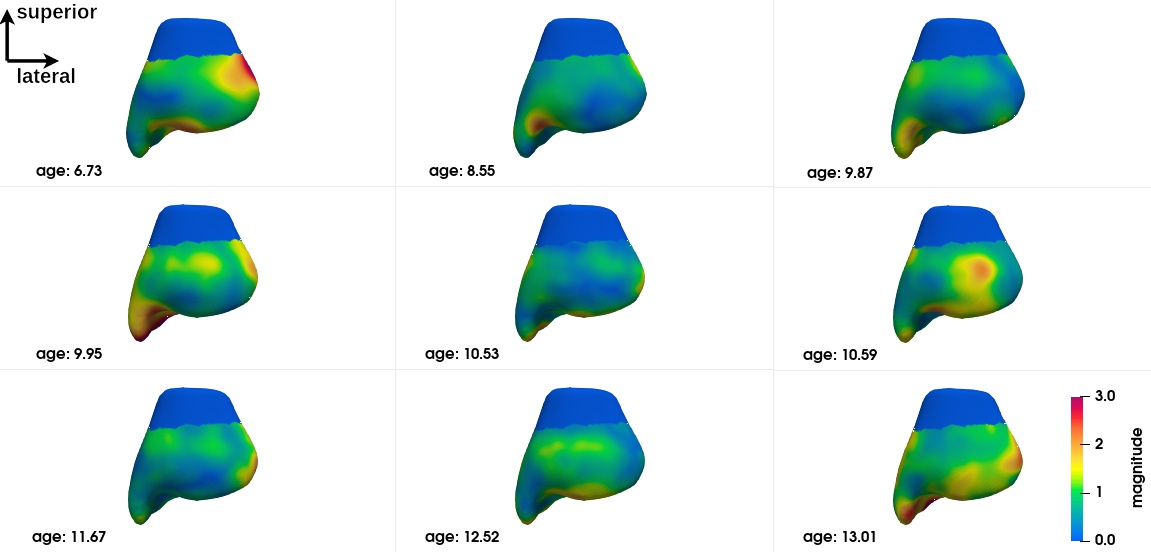

Supplement: Supplementary file 1 [file DataSheet1.zip › supplementary/Image 11.JPEG]

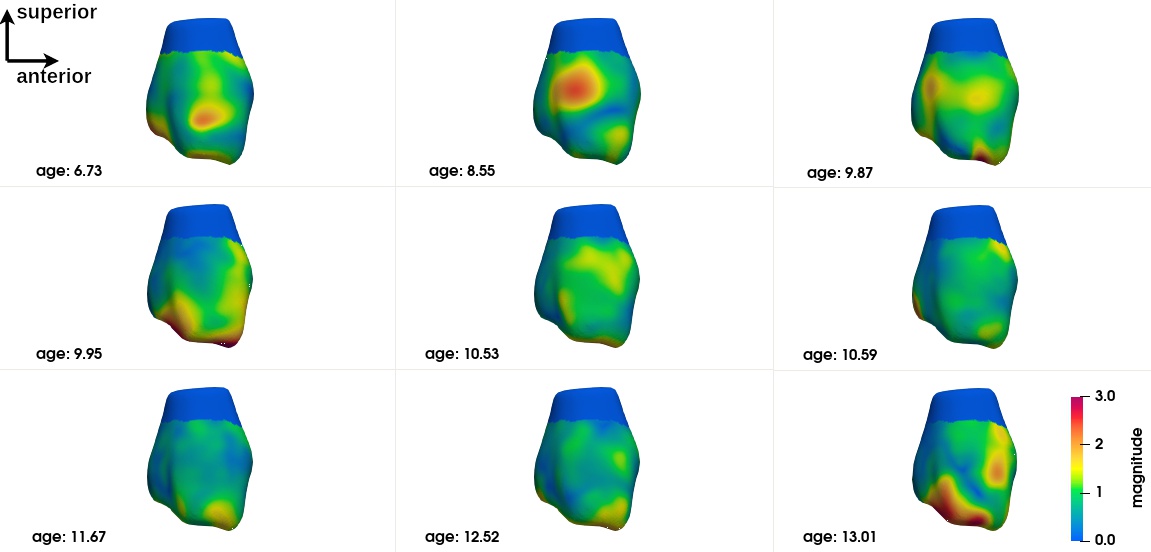

Supplement: Supplementary file 1 [file DataSheet1.zip › supplementary/Image 13.JPEG]

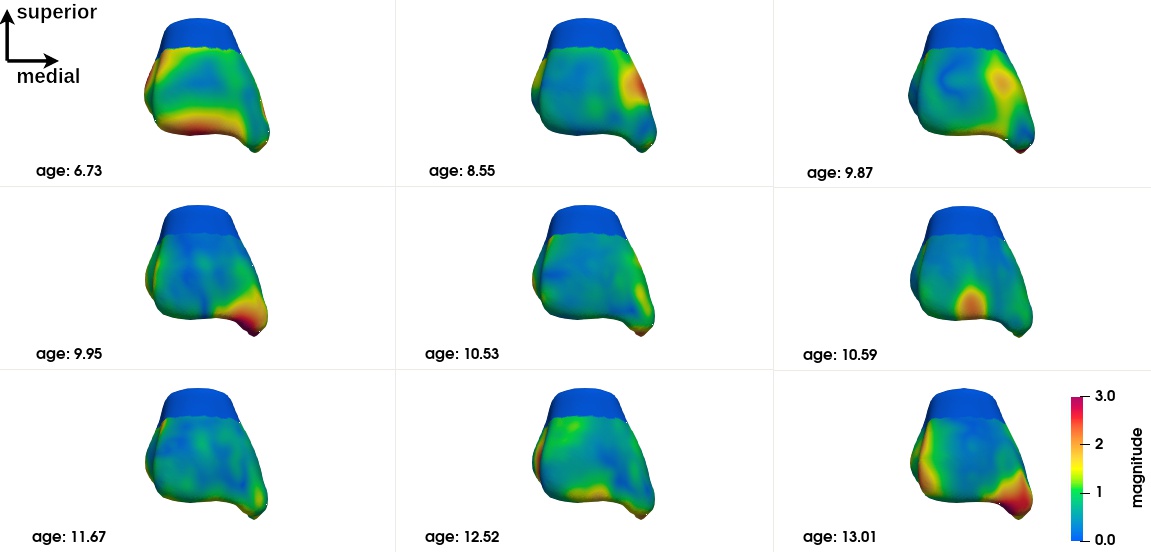

Supplement: Supplementary file 1 [file DataSheet1.zip › supplementary/Image 14.JPEG]
